# Gut, oral, and nasopharyngeal microbiota dynamics in the clinical course of hospitalized infants with respiratory syncytial virus bronchiolitis

**DOI:** 10.3389/fcimb.2023.1193113

**Published:** 2023-08-23

**Authors:** Sara Roggiani, Daniele Zama, Federica D’Amico, Alessandro Rocca, Marco Fabbrini, Camilla Totaro, Luca Pierantoni, Patrizia Brigidi, Silvia Turroni, Marcello Lanari

**Affiliations:** ^1^ Microbiomics Unit, Department of Medical and Surgical Sciences, University of Bologna, Bologna, Italy; ^2^ Paediatric Emergency Unit, Istituto di Ricovero e Cura a Carattere Scientifico (IRCCS) Azienda Ospedaliero-Universitaria Di Bologna, Bologna, Italy; ^3^ Specialty School of Pediatrics, University of Bologna, Bologna, Italy; ^4^ Unit of Microbiome Science and Biotechnology, Department of Pharmacy and Biotechnology, University of Bologna, Bologna, Italy

**Keywords:** respiratory syncytial virus, gut microbiota, oral microbiota, nasopharyngeal microbiota, infants (0 to 24 months)

## Abstract

**Introduction:**

Respiratory syncytial virus (RSV) is the most common cause of bronchiolitis and hospitalization in infants worldwide. The nasopharyngeal microbiota has been suggested to play a role in influencing the clinical course of RSV bronchiolitis, and some evidence has been provided regarding oral and gut microbiota. However, most studies have focused on a single timepoint, and none has investigated all three ecosystems at once.

**Methods:**

Here, we simultaneously reconstructed the gut, oral and nasopharyngeal microbiota dynamics of 19 infants with RSV bronchiolitis in relation to the duration of hospitalization (more or less than 5 days). Fecal samples, oral swabs, and nasopharyngeal aspirates were collected at three timepoints (emergency room admission, discharge and six-month follow-up) and profiled by 16S rRNA amplicon sequencing.

**Results:**

Interestingly, all ecosystems underwent rearrangements over time but with distinct configurations depending on the clinical course of bronchiolitis. In particular, infants hospitalized for longer showed early and persistent signatures of unhealthy microbiota in all ecosystems, i.e., an increased representation of pathobionts and a depletion of typical age-predicted commensals.

**Discussion:**

Monitoring infant microbiota during RSV bronchiolitis and promptly reversing any dysbiotic features could be important for prognosis and long-term health.

## 1 Introduction

Bronchiolitis, a common lower respiratory tract infection in infants, has a major impact on the health-care system being the most frequent cause of hospitalization in children under five years of age (Hall et al., 2009; Shi et al., 2017). Respiratory syncytial virus (RSV) is the most common causative agent of bronchiolitis, accounting for 33,1 million lower respiratory tract infections worldwide in early childhood in 2015 (Shi et al., 2017; Mazur et al., 2018). A variety of clinical manifestations of RSV bronchiolitis are recorded, ranging from an asymptomatic or mild course of infection, characterized by rhinorrhea, fever, cough, and respiratory distress, to a severe course that might require hospitalization, oxygen therapy, positive pressure ventilation support and, if necessary, admission to the pediatric intensive care unit (Meissner, 2016). At worst, it could lead to death; it was estimated that globally in 2015 there were around 27,300 in-hospital deaths due to RSV-associated acute lower respiratory tract infections in infants younger than 6 months (Shi et al., 2017; Mazur et al., 2018).

Age appears to be one of the major factors influencing the extent of the disease, with infants under 6 months being more frequently affected by severe bronchiolitis (Meissner, 2016). Other risk factors include prenatal exposure to tobacco smoke, outdoor air pollution, prematurity, low birth weight, lack of breastfeeding, cardiopulmonary disease and immunodeficiencies (Hall et al., 2009; Lanari et al., 2015a, Lanari et al., 2015b, Figueras-Aloy et al., 2016). Emerging evidence suggests that microorganisms inhabiting the respiratory tract might play a role in influencing the clinical course after the bronchiolitis event (Fujiogi et al., 2022). For example, the dominance of different strains, as *Streptococcus*, *Haemophilus* and *Moraxella* in the nasopharyngeal microbiota (NM) has already been established playing a role in the context of viral respiratory tract infections in infants, including RSV bronchiolitis (Rossi et al., 2021). Shifts in NM composition at the time of viral infection may enhance the severity of RSV infection and delay viral clearance (Gulraiz et al., 2015; Mansbach et al., 2019). Moreover, the increased presence of *Haemophilus*, *Moraxella*, and *Streptococcus* has been associated with an increased risk of developing childhood asthma and recurrent wheezing (Rosas-Salazar et al., 2018; Raita et al., 2022), known to be two of the most frequent long-term consequences of RSV infection in infants (Stein et al., 1999; Blanken et al., 2013). On the other hand, the role of other host-associated microbial ecosystems in the pathogenesis of RSV bronchiolitis has not yet been elucidated. To date, in fact, only a few studies have explored the oral microbiota (OM) in relation to RSV infection in infants. Although diseased infants showed a *Streptococcus*-dominant OM profile like healthy ones, higher relative abundances of *Haemophilus*, *Moraxella* and *Klebsiella* have been suggested to contribute to the development of subsequent recurrent wheezing (Hu et al., 2017; Zhang et al., 2020). Regarding the gut microbiota (GM), it was observed that infants characterized by a profile dominated by *Bacteroides* were subject to a higher likelihood of bronchiolitis, unlike those with an *Enterobacter*/*Veillonella*-dominant profile (Hasegawa et al., 2017). However, most of the available studies have focused on a single timepoint and, to the best of our knowledge, none has investigated all three ecosystems at once.

To confirm and extend the above knowledge, here we conducted a pilot study on 19 pediatric patients under one year of age, enrolled upon admission to the emergency room and confirmed diagnosis of RSV bronchiolitis. Specifically, we profiled GM, OM, and NM by 16S rRNA amplicon sequencing at three different timepoints (hospitalization to the emergency room, discharge, and six-month follow-up). For each ecosystem, microbiota dynamics were reconstructed with the aim of verifying the association with the clinical course of bronchiolitis. In addition to assessing the impact of RSV bronchiolitis on the three microbial ecosystems, our study identified potential microbial signatures related to the duration of hospitalization.

## 2 Materials and methods

### 2.1 Patient enrolment and sampling

This is a pilot study evaluating RSV-positive patients enrolled in a prospective monocentric longitudinal observational study conducted at the Pediatric Emergency Department of Unit, IRCCS Azienda Ospedaliero-Universitaria in Bologna, Italy. This study was ethically approved by the Area Vasta Emilia Centro Ethics Committee (reference 737/2018/Sper/AOUBo). Inpatient infants with a first clinical diagnosis of acute bronchiolitis between September 2021 and April 2022 were included. The diagnosis was based on findings of respiratory distress syndrome characterized by wheezing and/or rales on chest auscultation and signs of upper and lower respiratory tract infection. We included infants younger than one year, in agreement with the Italian guidelines for acute bronchiolitis (Baraldi et al., 2014), after obtaining parental consent to participation. We excluded children older than one year, those previously admitted to the hospital with a diagnosis of acute bronchiolitis, and those with severe chronic diseases like congenital cardiopulmonary disease, muscular dystrophy, cystic fibrosis, congenital or acquired immunodeficiency, and other conditions that may cause respiratory distress. For each enrolled infant, the following data were collected: i) personal and anamnestic data (including sex, date of birth, age, ethnicity, gestational age, mode of delivery, birth weight, breastfeeding, and weight at hospitalization); ii) hospitalization data (hospitalization date, discharge date, length of stay, admission to Pediatric Intensive Care Unit); iii) clinical data (duration of symptoms prior to admission, vitals at hospitalization (heart rate, respiratory rate, body temperature, oxygen saturation), signs of respiratory distress (increased respiratory rate, retractions), wheezing and/or rales, presence of decreased oral intake, complications); and iv) treatment data (drugs administrated, respiratory support duration).

**Figure 1:**
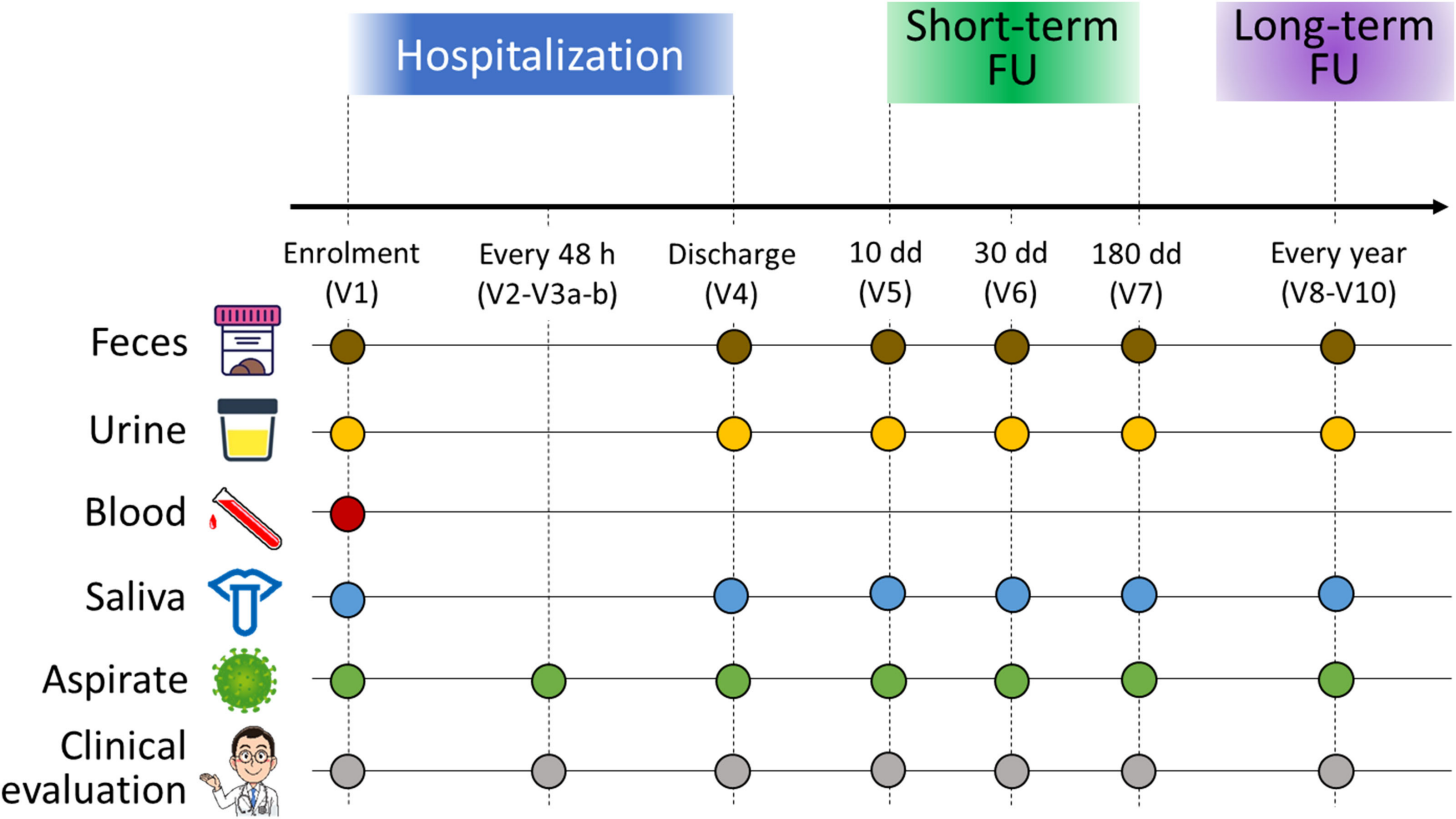
Study design and sample collection scheme. For each infant enrolled in the study, feces (brown dots), urine (yellow dots), blood (red dot), oral swabs (blue dots) and nasopharyngeal aspirates (green dots) were collected. All infants underwent clinical evaluation (gray dots). Enrolled infants were sampled over time: at admission to the emergency room (V1), every 48 hours (V2-V3 a-b), at discharge (V4), and then at 10 (V5), 30 (V6), and 180 (V7) days after discharge for a short-term follow-up. A long-term follow-up was foreseen in the study design, consisting of sampling every year up to three years of age. In this work, we examined only the timepoints V1, V4 and V7.

The study was divided into three phases (hospitalization, short-term follow-up, and long-term follow-up) following the scheme in **Figure 1**. According to the study design, blood (for assessing the inflammatory state of the host), nasopharyngeal aspirates (for assessing the viral load, the dosage of cytokines, the inflammatory infiltrate, and the NM profiling), oral swabs (for OM profiling), urine (for metabolome analysis) and fecal samples (for GM profiling) were collected from each patient. During the hospitalization phase, which lasted from admission to the emergency room to discharge, biological samples were collected at different timepoints: at enrolment (V1), every 48 hours (V2-V3 a-b), and discharge (V4). For short-term follow-up, infants were sampled at 10, 30 and 180 days after discharge (timepoint V5, V6, and V7, respectively). Then, patients will be called back for periodic visits every year for medical history and for the collection of samples up to three years of age (timepoints from V8 to V10).

In this work, only nasopharyngeal aspirates, oral swabs, and stool samples collected at emergency room admission (V1), discharge (V4) and six-month follow-up (V7) were analyzed to characterize NM, OM, and GM, respectively, as described below.

### 2.2 Microbial DNA extraction from stools, oral swabs, and nasopharyngeal aspirates

Microbial DNA was extracted from feces using the repeated bead-beating plus column method (Yu and Morrison, 2004), with a few minor adjustments as previously described (D’Amico et al., 2019). Approximately 250 mg of stool sample was suspended in 1 mL of lysis buffer (500 mM NaCl, 50 mM Tris-HCl pH 8, 50 mM EDTA, and 4% sodium dodecyl sulphate) and subjected to three 1-min cycles in a FastPrep instrument (MP Biomedicals, Irvine, CA, USA) at 5.5 movements/s, in the presence of four 3-mm glass beads and 0.5 g of 0.1-mm zirconia beads. After an incubation step at 95°C for 15 min and centrifugation at 13,000 rpm for 5 min, 260 µL of 10 M ammonium acetate was added to the supernatant, followed by a 5-min incubation on ice and a 10-min centrifugation at 13,000 rpm. Nucleic acids were precipitated by incubation on ice with one volume of isopropanol for 30 min. The nucleic acids pellets thus obtained were washed with 70% ethanol, re-suspended in 100 µL of TE (10 mM Tris-HCl, 1 mM EDTA pH 8.0) buffer, and treated with 2 µL of 10 mg/mL DNase-free RNAse at 37°C for 15 min. The samples were then incubated with 15 µL of proteinase K and 200 µL of AL buffer for 10 min at 70°C. DNA purification was achieved with the DNeasy Blood and Tissue Kit (QIAGEN, Hilden, Germany) according to the manufacturer’s instructions. DNA concentration and quality were evaluated using a NanoDrop ND-1000 spectrophotometer (NanoDrop Technologies,Wilmington, DE, USA).

For oral swabs, the cotton swab was suspended in 1 mL of saline and vortexed for 2 min. The swabs were removed, and the solution was centrifuged at 8,000 × *g* for 10 min at 4°C. For nasopharyngeal aspirates, 1 mL of sample was centrifuged at 8,000 × *g* for 15 min at 4°C. For both types of samples, the supernatant was discarded, the pellet was resuspended in 180 µL of enzymatic lysis buffer (20 mM Tris-HCl pH 8.0, 2 mM sodium EDTA, 1.2% Triton^®^ X-100, and 20 mg/mL lysozyme) and incubated at 37°C for 30 min. After the addition of 0.2 g of 0.1-mm zirconia beads, the samples were vortexed for 1 min. Twenty-five µL of proteinase K and 200 µL of buffer AL were added to each sample and incubated at 56°C for 30 min. The samples were then treated according to the DNeasy Blood and Tissue Kit (QIAGEN) instructions as above.

### 2.3 16S rRNA gene amplification and Illumina MiSeq sequencing

Library preparation was performed for all samples as described in the Illumina protocol “16S Metagenomic Sequencing Library Preparation” (Illumina, San Diego, CA, USA). The V3–V4 hypervariable region of the 16S rRNA gene was amplified using the 341F and 785R primers containing Illumina adapter overhang sequences as previously reported (Klindworth et al., 2013). Amplification was performed with KAPA HiFi HotStart ReadyMix (Roche, Basel, Switzerland), setting the following thermocycle: 3 min at 95°C, 25 cycles of 30 s at 95°C, 30 s at 55°C, and 30 s at 72°C, and a final 5-min step at 72°C. An extraction negative control was processed along with the samples, as per laboratory practice. Amplicons were purified with a magnetic bead-based clean-up system (Agencourt AMPure XP; Beckman Coulter, Brea, CA, USA). Indexed libraries were prepared by limited-cycle PCR using Nextera technology, followed by a second clean-up step as described above. The final library, prepared by pooling samples to an equimolar concentration of 4 nM, was denatured and diluted to 5 pM with a 20% PhiX control. Sequencing was performed on an Illumina MiSeq platform using a 2 × 250 bp paired-end protocol, as per manufacturer’s guidelines.

### 2.4 Bioinformatics and statistical analysis

Raw sequence data were analyzed using a pipeline combining PANDAseq (Masella et al., 2012) and QIIME 2 (Bolyen et al., 2019). All sequences were filtered for length (minimum/maximum = 350/550 bp) and quality (default parameters). Next, the DADA2 pipeline was used to bin the remaining reads into amplicon sequence variants (ASVs). Taxonomic classification was performed using the VSEARCH algorithm against the Greengenes database (May 2019 release). The resulting ASV tables were used for computing ecological indices of alpha and beta diversity. Alpha diversity was evaluated using the Shannon index and Faith’s Phylogenetic Diversity (PD whole tree). Beta diversity was estimated by computing weighted and unweighted UniFrac distances, which were used to build principal coordinates analysis (PCoA) graphs. Multivariate associations between microbial profiles and clinical metadata (including length of hospitalization and clinical infection score across timepoints) were assessed using MaAsLin2 (Mallick et al., 2021), with “LM” analysis method, no normalization, no value transformation, and correcting *p* values with the Benjamini-Hochberg method. Only significant associations (*p* < 0.05) were considered and commented. In addition, we used the splinectomeR R package (Shields-Cutler et al., 2018) to further explore associations between longitudinal shifts in relative abundance of detected taxa and the length of hospitalization or clinical infection score. This package reconstructs longitudinal splines taking into account the full trajectory of the data and highlighting differences in group trends against a random permutation of the data to simulate background noise. In detail, the relative abundances of each taxon detected were tested for differences in trends comparing groups based on hospitalization length (less than or equal to 5 days vs. more than 5 days, see paragraph “3.1 Study cohort description” in the Results section for patent stratification) and clinical infection score (mild vs. moderate, see **Table 1** for cutoff level). Following the developer’s instructions, data were tested with permutations to check for non-zero trends among a set of individuals over time, resulting in a set of permuted datapoints as well as a *p* value for the overall difference between the trends of the groups. Differences between groups were tested longitudinally using the *sliding_spliner* function to identify the timepoints of greater divergence, and the resulting plots were produced using the *sliding_spliner.plot.splines* function.

**Table 1:** Demographic and clinical data of the enrolled cohort.

	≤ 5 days (n=11)	> 5 days (n=8)	Total (n=19)	p-value
Age				
**-** Average, weeks **-** Median, weeks **-** ≤ 24 weeks, n (%) **-** > 24 weeks, n (%)	20.1 14.6 9 (82) 2 (18)	8.4 6.2 8 (100) 0	15.2 12 17 (89) 2 (11)	0.49
Weight, kg	6.27	5.23	5.82	
Sex, n (%)				
**-** Male **-** Female	6 (55) 5 (45)	6 (75) 2 (25)	12 (63) 7 (37)	0.63
Ethnicity, n (%)				
**-** Caucasian **-** Asiatic	9 (82) 2 (18)	8 (100) 0	17 (89) 2 (11)	0.49
Prematurity, n (%)	0	1 (13)	1 (5)	0.42
Vaginal delivery, n (%)	8 (63)	6 (75)	14 (74)	1
Cesarean delivery, n (%)	3 (27)	2 (25)	5 (26)	
Vaccinated, n (%)	7 (64)	2 (25)	9 (47)	0.17
Frequenting daycare/nursery, n (%)	2 (18)	0	2 (11)	0.49
Passive smoking exposure, n (%)	3 (27)	1 (13)	4 (21)	0.60
Maternal smoking during pregnancy, n (%)	0	0	0	1
Breastfeeding, n (%) **-** Ongoing	9 (82) 5 (45)	8 (100) 7 (88)	17 (89) 12 (63)	0.49 0.29
O_2_ saturation ≤ 94%, n (%)	4 (36)	2 (25)	6 (32)	0.66
Clinical score, > 4	5 (45)	2 (25)	7 (37)	0.63
RSV, n (%)	11 (100)	8 (100)	19 (100)	1
Other virus, n (%)	0	0	0	1
Coinfections, n (%)	0	0	0	1
Symptoms duration before admission, days	3	4	3,4	
O_2_ therapy, n (%) **-** Nasal, n (%) **-** HFNC, n (%)	7 (64) 6/7 (86) 1/7 (14)	8 (100) 8/8 (100) 0/8	15 (79) 14/15 (93) 1/15 (7)	0.10 0.47 0.47
O_2_ therapy > 48 hours, n (%)	5/7 (71)	6/8 (75)	11/15 (79)	1
Poor feeding, n (%)	7 (64)	6 (75)	13 (68)	0.66
IV hydration, n (%)	7 (64)	7 (88)	14 (74)	0.34
Aerosol, n (%)	11 (100)	8 (100)	19 (100)	1
Adrenaline, n (%)	4 (36)	5 (63)	9 (47)	0.37
Steroids, n (%)	5 (45)	3 (38)	8 (42)	1
Antibiotics, n (%)	6 (55)	3 (38)	9 (47)	0.65

Data are shown for the total cohort and for patients stratified by length of stay (less than or equal to 5 days vs. greater than 5 days). Clinical score was based on respiratory rate, signs of respiratory distress, use of accessory respiratory muscles, and lung auscultation (if > 4, the patient experienced at least a “moderate” course of infection). “Vaccinated” refers to the standard array of vaccines received by the infants. HFNC, high-flow nasal cannula; IV, intravenous; RSV, respiratory syncytial virus.

All statistical analyses were performed with R software. PCoA plots were generated using the “vegan” package (version 2.6-2, https://cran.r-project.org/web/packages/vegan/index.html), and data separation was tested by a permutation test with pseudo-F ratio (function “Adonis” in “vegan”). Kruskal–Wallis tests followed by *post-hoc* Wilcoxon tests were used to assess differences in alpha diversity and relative taxon abundances at phylum, family and genus levels among groups, *i.e.*, for each microbial ecosystem over time. Genus-level heatmaps were built using the “made4” package (https://bioconductor.org/packages/release/bioc/html/made4.html) and “heatmap.2” function from the “gplots” package (https://cran.r-project.org/web/packages/gplots/index.html). Analyses were conducted on the total cohort and on patients stratified by length of hospital stay (greater than or less than/equal to 5 days). A focus on antibiotic use (*i.e.*, a comparison between patients who received antibiotics and those who did not) was also made for NM. *P* values were corrected for multiple comparisons using the Benjamini–Hochberg method where appropriate. A false discovery rate (FDR) ≤ 0.05 was considered statistically significant, while a *p* value between 0.05 and 0.1 was considered a trend.

Demographical and clinical variables were compared between groups of patients. Categorical variables were presented as frequencies. Continuous variables were presented as median (interquartile range, IQR) or mean ± standard deviation (SD), according to data distribution, verified for each variable by the Kolmogorov-Smirnov test. Data were compared using χ2, Mann-Whitney, Student t, or Kruskal-Wallis test as needed. Analyses were conducted using GraphPad Prism 8.0.1 version and a *p* value < 0.05 was considered statistically significant.

## 3 Results

### 3.1 Study cohort description

A population of 19 infants with RSV bronchiolitis met the inclusion criteria. Demographic and clinical data of the cohort are summarized in **Table 1**. They were 12 (63%) males and 7 (37%) females with a mean age of 15.2 ± 12.1 months and a mean weight of 5.8 (IQR: 4.6-6.9) kg at the time of admission. The study population included predominantly full-term subjects (95%), and 14 (74%) had a vaginal delivery. Twelve (63%) were breastfeeding on admission, and four (21%) were exposed to passive smoke at home. Regarding interventions, 79% of our patients required oxygen therapy and specifically, 93% of them received low-flow oxygen through nasal cannulas; the rest was treated with high-flow nasal cannula. No patient needed non-invasive or invasive ventilatory support, nor admission to the pediatric intensive care unit during hospitalization. Based on the clinical score, none of the patients experienced a “severe” course of infection. Median length of hospital stay was 5 days.

The patients were then stratified into two groups based on the length of hospital stay, *i.e.*, less than or equal to 5 days (n=11) and more than 5 days (n=8). No statistically significant differences were found between the two groups for the above variables. For each patient, stool and nasopharyngeal aspirate sampling and oral swabbing were performed at three timepoints, *i.e.*, emergency room entry, discharge, and six-month follow-up, for a total of 168 samples (56 for GM, 56 for OM, and 56 for NM). All samples were analyzed through 16S rRNA amplicon sequencing, yielding 3,682,710 high-quality reads (mean ± SD; 21,791.9 ± 7,455.5).

### 3.2 Gut, oral, and nasopharyngeal microbiota dynamics in infants with RSV bronchiolitis from hospital admission to 6-month follow-up

First, we assessed the impact of potential confounding factors on patients’ GM, OM and NM at baseline, *i.e.*, admission to the emergency room. According to the weighted UniFrac PCoA analysis (**Figure S1**), segregation was found only for the NM profiles in relation to antibiotic administration (PERMANOVA, *p* = 0.02) and cortisone administration (*p* = 0.04).

**Figure 2:**
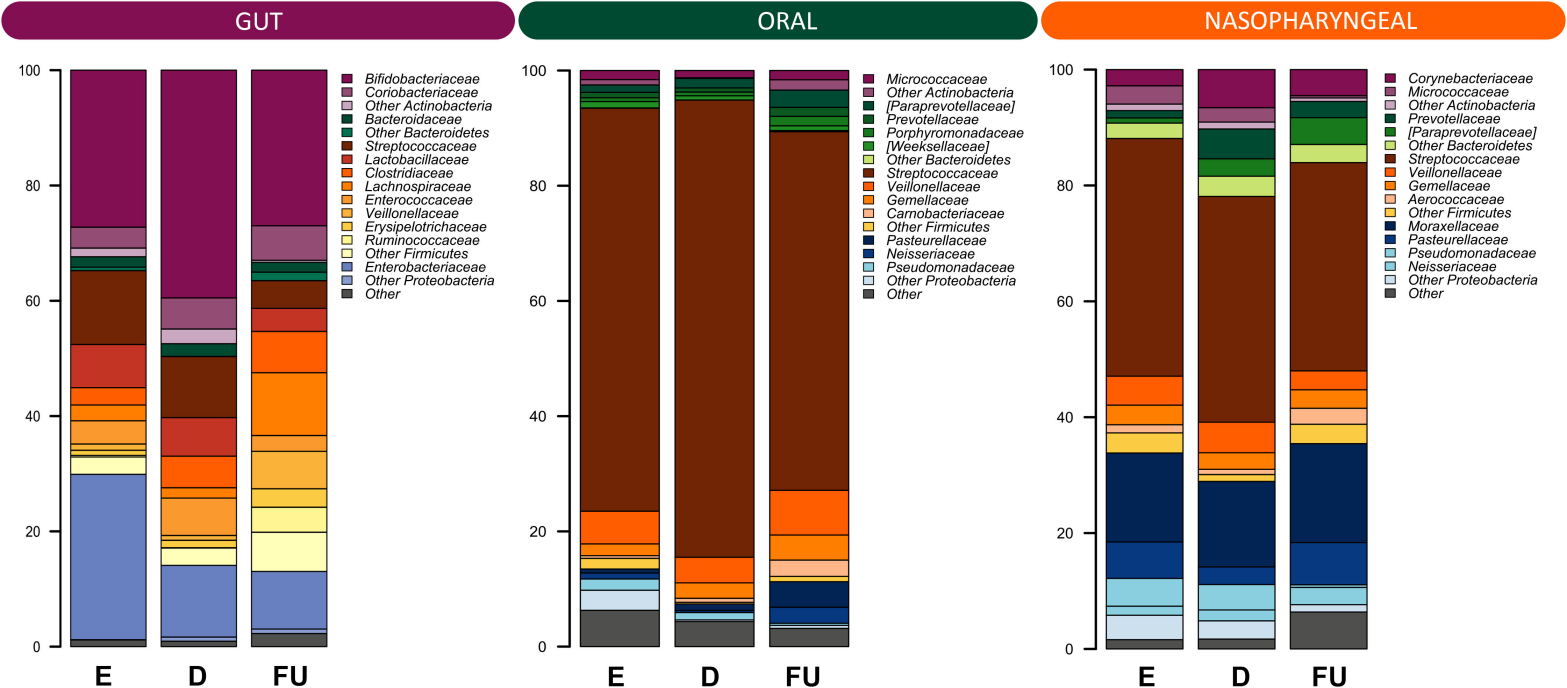
Family-level relative abundance profiles of the gut, oral and nasopharyngeal microbiota of infants with RSV bronchiolitis from emergency room admission to six months after discharge. Bar plots showing the relative abundance over time of the 12 most abundant families in each ecosystem. E, emergency room admission; D, discharge; FU, six-month follow-up.

Next, the dynamics of GM, OM and NM were reconstructed in the total cohort, from emergency room admission through discharge up to six-month follow-up. Alpha diversity increased gradually over time in all ecosystems with both metrics considered, *i.e.*, the Shannon index and Faith’s Phylogenetic Diversity (Wilcoxon test*, p* ≤ 0.02) (**Figure S2**). As for beta diversity (**Figure S3**), PCoA based on unweighted UniFrac distances showed segregation between fecal samples at follow-up and those collected at previous timepoints (PERMANOVA, *p* ≤ 0.05). Similarly, the structures of OM and NM differed at follow-up, as per unweighted UniFrac-based PCoA, from those of the respective samples collected at emergency room admission and discharge (*p* ≤ 0.03). Moreover, a segregation was detected between discharge and follow-up OM profiles in the PCoA based on weighted UniFrac distances (*p* = 0.01). **Figure 2** shows the family-level relative abundance profiles for all three microbial ecosystems. As for GM, Firmicutes (mean relative abundance ± standard error of the mean: 40.5% ± 3.9%), Actinobacteria (37.8% ± 4.0%) and Proteobacteria (17.7% ± 3.3%) dominated the baseline profile. The main families represented were *Bifidobacteriaceae* (31.3% ± 3.7%) and *Enterobacteriaceae* (17.1% ± 3.3%). At follow-up, we observed an increase in the relative abundance of Firmicutes, along with *Coriobacteriaceae*, *Clostridiaceae*, *Lachnospiraceae, Veillonellaceae, Ruminococcaceae, Peptostreptococcaceae*, and *Erysipelotrichaceae* (Wilcoxon test, *p* ≤ 0.04) (**Figure S4**). Furthermore, there was a trend towards decreased proportions of *Enterobacteriaceae* over time (*p* = 0.07). Some of these results were confirmed by applying the MaAsLin2 test, in particular for the families *Veillonellaceae*, *Peptostreptococcaceae*, *Lachnospiraceae*, and *Enterobacteriaceae* (**Table S1**).

OM was overall dominated by Firmicutes (82.0% ± 2.7%), specifically by the family *Streptococcaceae* (70.5% ± 3.1%). At follow-up, we observed an increase in the relative abundance of Bacteroidetes, Proteobacteria and Fusobacteria (*p* ≤ 0.01), and a decrease in that of Firmicutes (*p* = 0.005) (**Figure S5**). At the family level, a follow-up increase in *Actinomycetaceae*, *[Paraprevotellaceae], Gemellaceae, Carnobacteriaceae, Veillonellaceae, Fusobacteriaceae, Leptotrichiaceae, Neisseriaceae*, and *Pasteurellaceae* (*p* ≤ 0.04) was found, together with a decrease in *Streptococcaceae* (*p* = 0.002). With MaAsLin2 we were able to confirm the results for *Carnobacteriaceae*, *Leptotrichiaceae*, *Pasteurellaceae*, and *Gemellaceae* (**Table S1**).

As for NM, the ecosystem was dominated by phyla Firmicutes (50.7% ± 3.2%) and Proteobacteria (29.5% ± 3.3%), and by families *Streptococcaceae* (38.6% ± 3.2%) and *Moraxellaceae* (15.7% ± 3.1%). The proportions of Fusobacteria and Bacteroidetes increased at follow-up as well as those of *[Paraprevotellaceae]*, *Neisseriaceae* and *Pasteurellaceae, Aerococcaceae, Carnobacteriaceae, Leptotrichiaceae* and *Fusobacteriaceae* (*p* ≤ 0.04), while those of *Pseudomonadaceae* decreased (*p* ≤ 0.05) (**Figure S6**). By applying MaAsLin2, no statistically significant results were obtained.

### 3.3 Potential gut, oral, and nasopharyngeal microbiota signatures related to the duration of hospitalization

For each microbial ecosystem, the compositional dynamics were then evaluated by stratifying the patients into 2 groups based on the length of hospital stay (greater vs. less than or equal to 5 days).

#### 3.3.1 Gut microbiota

**Figure 3:**
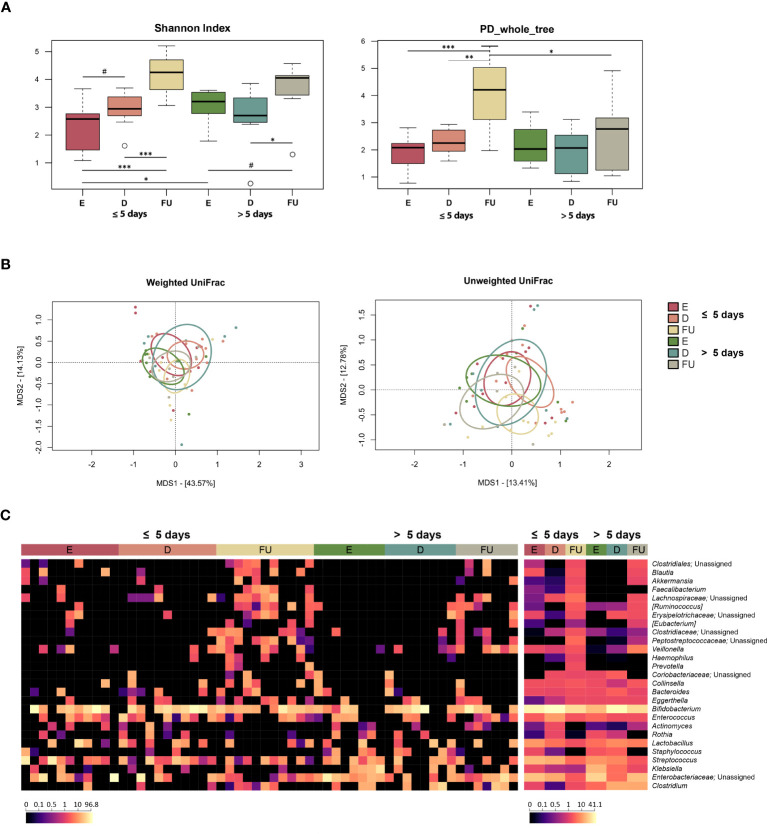
Gut microbiota dynamics in infants with RSV bronchiolitis with different length of hospitalization. **(A)** Boxplots showing the distribution of alpha diversity, computed with the Shannon index (left) and Faith’s Phylogenetic Diversity (PD_whole_tree; right), for fecal samples collected at the emergency room admission (E), discharge (D) and six-month follow-up (FU) from patients hospitalized for less than or equal to 5 days vs. more than 5 days. Wilcoxon test, * *p* value ≤ 0.05; ** *p* value ≤ 0.01; *** *p* value ≤ 0.001; # *p* value ≤ 0.09. **(B)** Principal Coordinates Analysis (PCoA) based on weighted UniFrac (left) and unweighted UniFrac (right) distances between microbiota profiles. A significant separation was found between D and FU samples only for infants hospitalized for less time with unweighted UniFrac (PERMANOVA, *p* = 0.03). **(C)** Heatmap of the relative abundances of the top 27 most abundant genera across all samples (left) and as mean values (right).

Alpha diversity increased gradually over time in both patient groups but was higher at baseline and lower at follow-up in those hospitalized for more than 5 days (with the Shannon index and Faith’s Phylogenetic Diversity, respectively) (Wilcoxon test, *p* ≤ 0.05) (**Figure 3A**). As for beta diversity, the unweighted UniFrac-based PCoA showed segregation between GM profiles at discharge and follow-up only for infants hospitalized for less time (PERMANOVA, *p* = 0.03) (**Figure 3B**; **Table S2**).

From the taxonomic point of view, many differences emerged, even at a high phylogenetic level (**Figures 3C**, **S7**). In particular, a decrease in Proteobacteria was observed at discharge in infants hospitalized for more than 5 days (Wilcoxon test, *p* = 0.03). Furthermore, the relative abundance of this phylum was higher at enrolment (*p* = 0.04) and tended to be higher at follow up (*p* = 0.08) in this group of patients than in infants hospitalized for less than or equal to 5 days. Consistently, these infants showed increased proportions of *Enterobacteriaceae* at enrolment, with follow-up values still higher than those of infants hospitalized for less time, although reduced compared with the initial values observed at emergency room admission (*p* ≤ 0.05). Conversely, the proportions of *Veillonella* increased at follow-up in infants hospitalized for more than 5 days (*p* ≤ 0.05), although the proportions of its family *Veillonellaceae* increased at follow-up in both groups of patients. On the other hand, infants hospitalized for less time showed an increase at follow-up in the proportions of *Coriobacteriaceae* (particularly *Eggerthella*), *Clostridiaceae*, *Ruminococcaceae* (particularly *Faecalibacterium*), *Pasteurellaceae*, *Blautia*, *[Ruminoccoccus]*, *[Eubacterium]*, and *Haemophilus* (*p* ≤ 0.05). *Erysipelotrichaceae*, *Lachnospiraceae*, and *Peptostreptococcaceae* increased at follow-up in both patient groups (*p* ≤ 0.05), but *Peptostreptococcaceae* tended to be much more represented in infants hospitalized for less time (*p* = 0.06). Using multivariate linear models with MaAsLin2 (**Figure S8**; **Table S1**), we were able to confirm the increased presence of *Peptostreptococcaceae, Pasteurellaceae*, and *Haemophilus* at follow-up in infants hospitalized for less than 5 days. In addition, an increased relative abundance of *Klebsiella* was detected at enrolment in infants hospitalized for more than 5 days.

The data were further explored using the splinectomeR R package, which highlighted that infants hospitalized for more than 5 days were characterized by increased abundance of *Clostridiaceae* and *Clostridium* at discharge and follow-up (*p* < 0.05) (**Figure S9**). Spline trends also confirmed increased proportions of *Klebsiella* at enrolment in this group of infants (*p* = 0.02), and increased proportions of *Peptostreptococcaceae* at follow-up in infants hospitalized for less than 5 days (*p* = 0.03). *Staphylococcaceae* and *Staphylococcus* appear to increase at discharge in infants hospitalized for more than 5 days (*p* ≤ 0.05). Regarding the associations with the clinical score, the only statistically significant difference was found for *Blautia*, whose relative abundance appeared to be higher at enrolment and follow-up in infants with a “moderate” course of infection (*p* = 0.01) (**Figure S10**).

#### 3.3.2 Oral microbiota

**Figure 4:**
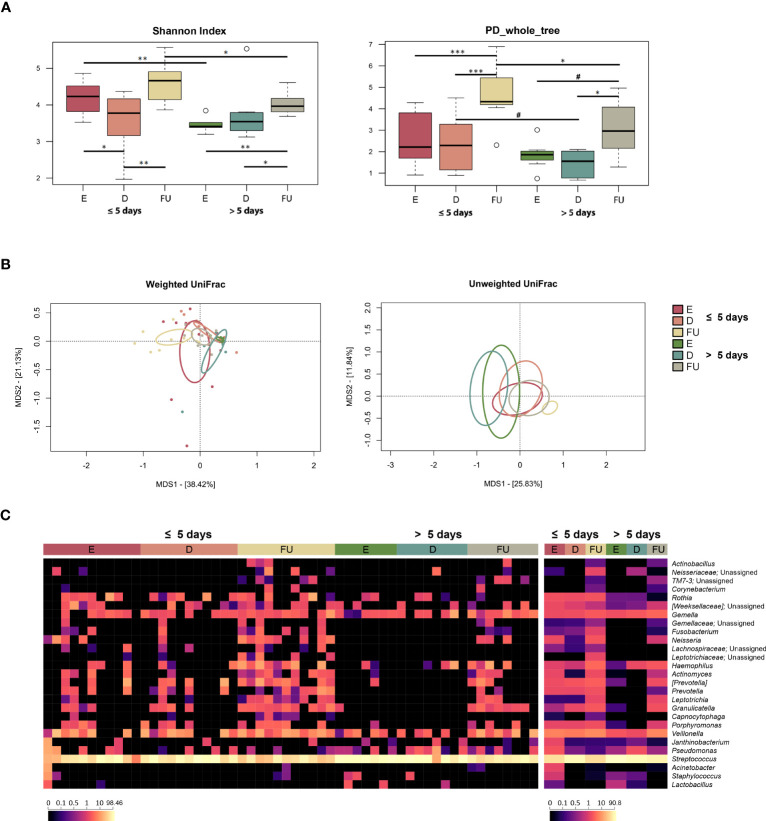
Oral microbiota dynamics in infants with RSV bronchiolitis with different length of hospitalization. **(A)** Boxplots showing the distribution of alpha diversity, computed with the Shannon index (left) and Faith’s Phylogenetic Diversity (PD_whole_tree; right), for oral swab samples collected at the emergency room admission (E), discharge (D) and six-month follow-up (FU) from patients hospitalized for less than or equal to 5 days vs. more than 5 days. Wilcoxon test, * *p* value ≤ 0.05; ** *p* value ≤ 0.01; *** *p* value ≤ 0.001; # *p* value ≤ 0.09. **(B)** Principal Coordinates Analysis (PCoA) based on weighted UniFrac (left) and unweighted UniFrac (right) distances between microbiota profiles. A significant separation was found over time within each patient group, as well as between the two patient groups for the E, D and FU samples (PERMANOVA, *p* ≤ 0.04). **(C)** Heatmap of the relative abundances of the top 27 most abundant genera across all samples (left) and as mean values (right).

Alpha diversity overall increased over time in both patient groups but was higher at both baseline and follow-up in those hospitalized for less time (Wilcoxon test, *p* ≤ 0.04) (**Figure 4A**). PCoA based on weighted UniFrac distances showed segregation between the two patient groups at these timepoints (PERMANOVA, *p* < 0.02) (**Figure 4B**; **Table S2**). A separation between follow-up profiles was also found with the unweighted UniFrac metric, which also highlighted a separation between the discharge profiles (*p* < 0.05). Moreover, in both groups of patients, significant differences were observed between OM structures over time (*p* ≤ 0.04).

From the taxonomic point of view, again many differences emerged, even at a high phylogenetic level (**Figures 4C**, **S11**). In particular, the increase in Proteobacteria observed at follow-up for the entire cohort specifically characterized infants hospitalized for less than or equal to 5 days (Wilcoxon test, *p* ≤ 0.004). Furthermore, the proportions of Proteobacteria in this patient group were higher than in the other group, at both baseline and follow-up (*p* ≤ 0.05). Infants hospitalized for less time also showed increased proportions of Firmicutes at discharge (*p* = 0.01), which decreased at follow-up (*p* = 0.001), as observed for the entire cohort, being lower compared to the other patient group at both enrolment and follow up (*p* ≤ 0.009). On the other hand, the phylum Bacteroidetes increased at follow-up only in infants hospitalized for more than 5 days (*p* < 0.03), still being lower than in the other group (*p* ≤ 0.04). Fusobacteria increased at follow-up in both patient groups (*p* ≤ 0.04). Even at lower phylogenetic levels, many changes were observed, including common and unique variations. As for the former, *Actinomycetaceae* (and its genus *Actinomyces*), *Gemellaceae*, *Carnobacteriaceae* (and *Granulicatella*), and *[Paraprevotellaceae]* (and *[Prevotella]*) increased or tended to increase at follow-up in both groups of patients (*p* ≤ 0.07). On the other hand, *Pasteurellaceae* and its genus *Haemophilus*, *Prevotellaceae* (and *Prevotella), Fusobacteriaceae* (and *Fusobacterium*), *Leptotrichiaceae* (and *Leptotrichia*), *Neisseriaceae* (and *Neisseria*) increased at follow-up only in infants hospitalized for less time (*p* < 0.05). Finally, *Streptococcaceae* family and *Streptococcus* genus were overrepresented at baseline in infants hospitalized for more than 5 days, decreased at follow-up in both patient groups, but their relative abundances remained significantly higher in those hospitalized longer (*p* ≤ 0.05). The increased relative abundances of *Leptotrichiaceae*, *Pasteurellaceae*, *Haemophilus*, *Neisseriaceae* and *Neisseria* at follow-up in infants hospitalized for less time, as well as the increased presence at enrolment of *Streptococcaceae* and *Streptococcus* in infants hospitalized for more than 5 days were confirmed using MaAsLin2 (**Figure S8**; **Table S1**).

According to the splinectomeR analysis, *Actinomycetaceae*, *Carnobacteriaceae*, *Granulicatella* and *Neisseria* increased at follow-up in both groups of infants, but with higher proportions in infants hospitalized for less than or equal to 5 days (*p* ≤ 0.05) (**Figure S9**). On the other hand, *Micrococcaceae*, *Prevotellaceae* and *Rothia* increased at follow-up only in patients hospitalized for less time (*p* ≤ 0.04). At enrolment and discharge, a statistically significant difference was found between infants hospitalized for less than or equal to 5 days and those hospitalized for more than 5 days with regard to *Veillonellaceae* (and its genus *Veillonella*), with higher values recorded in the former group, but this difference disappeared at follow-up (*p* ≤ 0.05). Regarding the associations with the clinical score (**Figure S10**), *Oxalobacteraceae* and its genus *Janthinobacterium* were enriched at enrolment in infants with a “moderate” course of infection (*p* ≤ 0.04). These infants also appeared to be characterized by an increased relative abundance at follow-up of *Leptotrichiaceae* unassigned, *Neisseriaceae*, and *Neisseria* (*p* ≤ 0.03). On the other hand, higher proportions of *Streptococcaceae* and *Streptococcus* were found at enrolment in infants with a “mild” course of infection compared to the other group of infants (*p* ≤ 0.04). According to MaAsLin2, infants with a “mild” course of infection were also characterized by lower proportions of *Pasteurellaceae* and *Haemophilus* at follow-up compared to “moderate” patients (*p* ≤ 0.001).

#### 3.3.3 Nasopharyngeal microbiota

**Figure 5:**
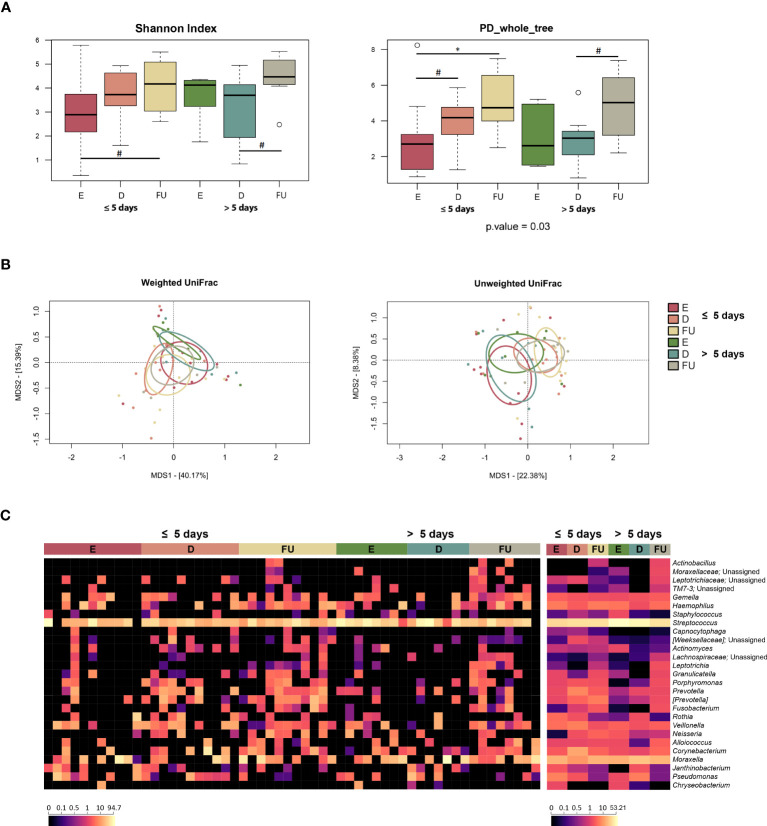
Nasopharyngeal microbiota dynamics in infants with RSV bronchiolitis with different length of hospitalization. **(A)** Boxplots showing the distribution of alpha diversity, computed with the Shannon index (left) and Faith’s Phylogenetic Diversity (PD_whole_tree; right) for nasopharyngeal aspirate samples collected at the emergency room admission (E), discharge (D) and six-month follow-up (FU) from patients hospitalized for less than or equal to 5 days vs. more than 5 days. Wilcoxon test, * *p* value ≤ 0.05; # *p* value ≤ 0.09. **(B)** Principal Coordinates Analysis (PCoA) based on weighted UniFrac (left) and unweighted UniFrac (right) distances between microbiota profiles. A separation was found over time within each patient group, as well as between the two patient groups for E and D samples (PERMANOVA, *p* ≤ 0.07). **(C)** Heatmap of the relative abundances of the top 27 most abundant genera across all samples (left) and as mean values (right).

Alpha diversity increased or tended to increase at follow-up in both patient groups (Wilcoxon test, *p* ≤ 0.09) (**Figure 5A**). As for beta-diversity, unweighted UniFrac-based PCoA showed segregation over time for the NM profiles of both groups of infants (PERMANOVA, *p* ≤ 0.04). Furthermore, we found a trend of separation between the NM structures of the two patient groups at enrolment with the weighted UniFrac metric and discharge with the unweighted UniFrac metric (*p* ≤ 0.07) (**Figure 5B**; **Table S2**).

From the taxonomic point of view (**Figures 5C**, **S12**), the increase at follow-up in *[Paraprevotellaceae]* (and its genus *[Prevotella]*) and *Neisseriaceae* (and *Neisseria*), observed for the entire cohort, specifically characterized infants hospitalized for less than or equal to 5 days (*p* ≤ 0.02). Moreover, the proportions of *Neisseriaceae* at enrolment and *[Paraprevotellaceae]* at follow-up tended to be greater than those of infants hospitalized for more than 5 days (*p* ≤ 0.09). Infants hospitalized for less time also showed increased relative abundances of *Prevotellaceae* (and *Prevotella*) and *Pasteurellaceae* (and *Haemophilus*) at follow-up (*p* ≤ 0.05). On the other hand, infants hospitalized for longer showed an increase in *Oxalobacteraceae* (particularly *Janthinobacterium*) at discharge, whose proportions then decreased at follow-up (*p* ≤ 0.04). It is also worth noting that *Staphylococcaceae, Streptococcaceae* and *Streptococcus* tended to be overrepresented at baseline in infants hospitalized longer (*p* ≤ 0.09). Finally, *Fusobacteriaceae* (and *Fusobacterium*) and *Leptotrichia* increased at follow-up in both patient groups (*p* < 0.05). MaAsLin2 analysis confirmed the increased relative abundance of *Leptotrichia* and its family *Leptotrichiaceae* in both patient groups at follow-up (**Figure S8**; **Table S1**). In contrast, when searching for associations with clinical score, we found a decrease in the proportions of *Leptotrichiaceae* and *Leptotrichia* at follow-up in both mild and moderate patients (p ≤ 0.001). No significant results were obtained using splinectomeR.

Given that a segregation was found between NM profiles at enrolment in relation to antibiotic administration, both groups of patients were further divided into two subgroups according to antibiotic exposure, and their taxonomic profiles at genus level were compared (**Figure S13** ***)***. The only significant difference was observed for the genus *Corynebacterium*, whose relative abundance was higher in infants receiving antibiotics and hospitalized for more than 5 days than in infants receiving antibiotics but hospitalized for a shorter period (*p* = 0.05). On the other hand, *Staphylococcus* and *Streptococcus* tended to be enriched in infants who did not receive antibiotics and were hospitalized for more than 5 days compared with infants who did not receive antibiotics but were hospitalized for a shorter period (*p* ≤ 0.09). *Veillonella* also tended to be enriched in infants who did not receive antibiotics and were hospitalized for more than 5 days compared to those who received antibiotics (*p* = 0.08), while the opposite trend was observed for *Alloiococcus*, *i.e.*, it tended to be enriched in infants receiving antibiotics and hospitalized for more than 5 days compared to those who did not receive antibiotics (*p* = 0.08).

## 4 Discussion

For the first time, in this pilot study, the temporal dynamics of three different host-associated microbial ecosystems (*i.e.*, fecal, oral, and nasopharyngeal) were explored simultaneously in infants with RSV bronchiolitis, from emergency room admission to 6 months after discharge. Such dynamics were reconstructed separately in patients with different duration of hospitalization, to study the association with the natural history of the disease. In addition, several methods have been used to evaluate differences in such dynamics, providing more robust results overall. In line with the available literature (Hasegawa et al., 2016a; Hasegawa et al., 2016b; Hu et al., 2017; Stewart et al., 2017; Harding et al., 2020), all the ecosystems investigated underwent rearrangements over time but with distinct configurations depending on the clinical course of bronchiolitis.

Regarding GM, the greatest fluctuations were observed in infants hospitalized for less time. In particular, these infants showed a gradual increase in a number of age-predicted microorganisms, such as *Coriobacteriaceae* (particularly *Eggerthella*), *Ruminococcaceae* (including *Faecalibacterium*) and *Blautia*, suggesting the recovery of an overall eubiotic GM profile. Indeed, these are typically members of the developing infant microbiota and associated with its health (Leylabadlo et al., 2020; Liu et al., 2021; Roswall et al., 2021; Ou et al., 2022). Conversely, infants hospitalized longer showed an overall higher relative abundance of opportunistic pathogens, *i.e.*, *Enterobacteriaceae*, *Klebsiella* and *Clostridium* (Plata et al., 2009; Logan and Weinstein, 2017), since admission to the emergency room. Interestingly, in some patients, *Enterobacteriaceae* nearly dominated the GM (with relative abundance up to 96.3% at baseline) and their proportions remained high even in the follow-up, indicating the persistence of potentially harmful dysbiotic features. It must be said that their persistent overrepresentation may also have been fostered by the prolonged oxygen therapy to which all patients hospitalized for more than 5 days were subjected, resulting in an intestinal environment more favorable to facultative anaerobes such as enterobacteria (Rivera-Chávez et al., 2017).

Also with regard to OM, over the study period, the infants hospitalized for less time tended to acquire a eubiotic profile (compatible with age), while the microbiota of those hospitalized for longer was overall more stable and resilient, carrying on potentially unhealthy signatures up to 6 months after discharge. In particular, several taxa normally present in the oral niche of infants, such as *Haemophilus*, *Prevotella*, *Fusobacterium*, *Neisseria* and *Leptotrichia* (De Steenhuijsen Piters et al., 2015; Oba et al., 2020; D’Agostino et al., 2022), were much more represented at follow-up in infants hospitalized for less time. Conversely, those hospitalized for more than 5 days showed a peculiar enrichment (sometimes monodominance) in *Streptococcus*, a genus that includes species with pathogenic potential (Yumoto et al., 2019), at all timepoints, with relative abundance values up to 87.3% even at follow-up.

As for NM, the most characterized host-associated microbial ecosystem to date in the context of RSV bronchiolitis (De Steenhuijsen Piters et al., 2016; Hasegawa et al., 2016b; Hasegawa et al., 2017; Stewart et al., 2017; Mansbach et al., 2019), we found increased relative abundance over time of taxa such as *Neisseria*, *[Prevotella]*, *Haemophilus*, and *Prevotella*, in infants hospitalized for less time. Again, these are typical commensal bacteria of the infant NM (De Steenhuijsen Piters et al., 2015; Rocafort et al., 2021; Tozzi et al., 2021), which further strengthens the hypothesis of a rapid recovery of microbiota health. In contrast, infants hospitalized for more than 5 days showed no drastic changes in NM composition over time, with the exception of the increase at discharge of *Oxalobacteraceae* and its genus *Janthinobacterium*, suggesting the persistence of non-eubiotic characteristics for this ecosystem as well. This increase in *Janthinobacterium* in the nasopharynx of infants with respiratory tract infections is consistent with the available literature (Man et al., 2019). Furthermore, it should be noted that the proportions of *Staphylococcus* and *Streptococcus* tended to be high at baseline in these patients (with a trend to be more present in infants who did not receive antibiotics), paralleling what was found in the OM. Although with due caution, this could suggest the relevance of these microorganisms as an early biomarker of not only altered but also recalcitrant (*i.e.*, more difficult to manipulate) microbiota.

Our work had some limitations, including the lack of a control group. Nonetheless, the aim of the present study was to find microbiota signatures from different body sites related to the duration of hospitalization, and therefore to the clinical course of bronchiolitis, and not to disease development. Furthermore, we used the duration of hospitalization as a criterion to stratify patients, as none of the 32 available clinical scores for bronchiolitis were found to be sufficiently reliable to estimate the severity of hospitalized patients (Rodriguez-Martinez et al., 2018). In particular, the clinical score adopted in this study did not correlate with other outcomes of clinical severity (such as length of hospitalization, oxygen therapy and duration of oxygen therapy), which explains why this parameter was not used to classify patients, but the length of recovery was preferred. Another limitation is certainly the small sample size but only patients with an almost complete sampling trajectory for all three ecosystems were included. The main reasons for the lack of samples were patient and/or parent/guardian compliance, the severity of the disease that sometimes interfered with sampling, and in other cases the collection of insufficient biological material for analysis. Finally, it must be said that the infant microbiome changes over time and is generally characterized by higher plasticity and less stability than that of adults. Although some microbial features are undoubtedly unhealthy or dysbiotic (such as the overrepresentation of *Enterobacteriaceae* in the gut, *Streptococcus* in the mouth, and *Staphylococcus* and *Streptococcus* in the nasopharynx), we cannot rule out that other features are simply related to advancing age.

## 5 Conclusions

In this pilot study, we identified the compositional features of three different host-associated microbial ecosystems that potentially discriminated for clinical course of RSV bronchiolitis in infants. In particular, infants hospitalized for longer showed early and persistent signatures of unhealthy microbiota, *i.e.*, an increased representation of pathobionts and a depletion of typical commensals. In light of the relevance of a eubiotic developmental trajectory of the infant microbiota to long-term health (Cox et al., 2014), such features should be closely monitored and promptly reversed through appropriate manipulation strategies, to improve clinical management and reduce the risk of future complications (e.g., asthma and recurrent wheezing). Our findings should be validated in larger cohort studies, involving longer-term sampling and other omics approaches (e.g., metagenomics and metabolomics) for high-resolution taxonomic profiling (down to species level) and functional insights, to improve the identification of dysbiotic features at this critical stage of infant development. In this regard, the original study design provides for an extension of the cohort with subjects recruited in the coming epidemic seasons and the analysis of samples in the long-term follow-up, up to three years of age.

## Data availability statement

The data presented in the study are deposited in the NCBI repository, accession number PRJNA978360.

## Ethics statement

The studies involving humans were approved by the Area Vasta Emilia Centro Ethics Committee (reference 737/2018/Sper/AOUBo). The studies were conducted in accordance with the local legislation and institutional requirements. Written informed consent for participation in this study was provided by the participants’ legal guardians/next of kin.

## Author contributions

Conceptualization, ML and PB; Data Curation, SR, FD’A, DZ, AR and CT; Formal Analysis, SR, FD’A, MF, DZ and AR; Investigation, SR, FD’A, DZ and AR; Resources, PB; Supervision, PB; Visualization, SR and DZ; Writing—Original Draft, SR; Writing—Review and Editing, FD’A, ML, DZ and ST. All authors have read and agreed to the published version of the manuscript.
