# Supplementary material for: Gut, oral, and nasopharyngeal microbiota dynamics in the clinical course of hospitalized infants with respiratory syncytial virus bronchiolitis
Source: Front Cell Infect Microbiol. 2023 Aug 23;13:1193113. doi: 10.3389/fcimb.2023.1193113 (PMC10482328; doi:10.3389/fcimb.2023.1193113)
Supplement: Supplementary file 1 [file DataSheet_1.docx]

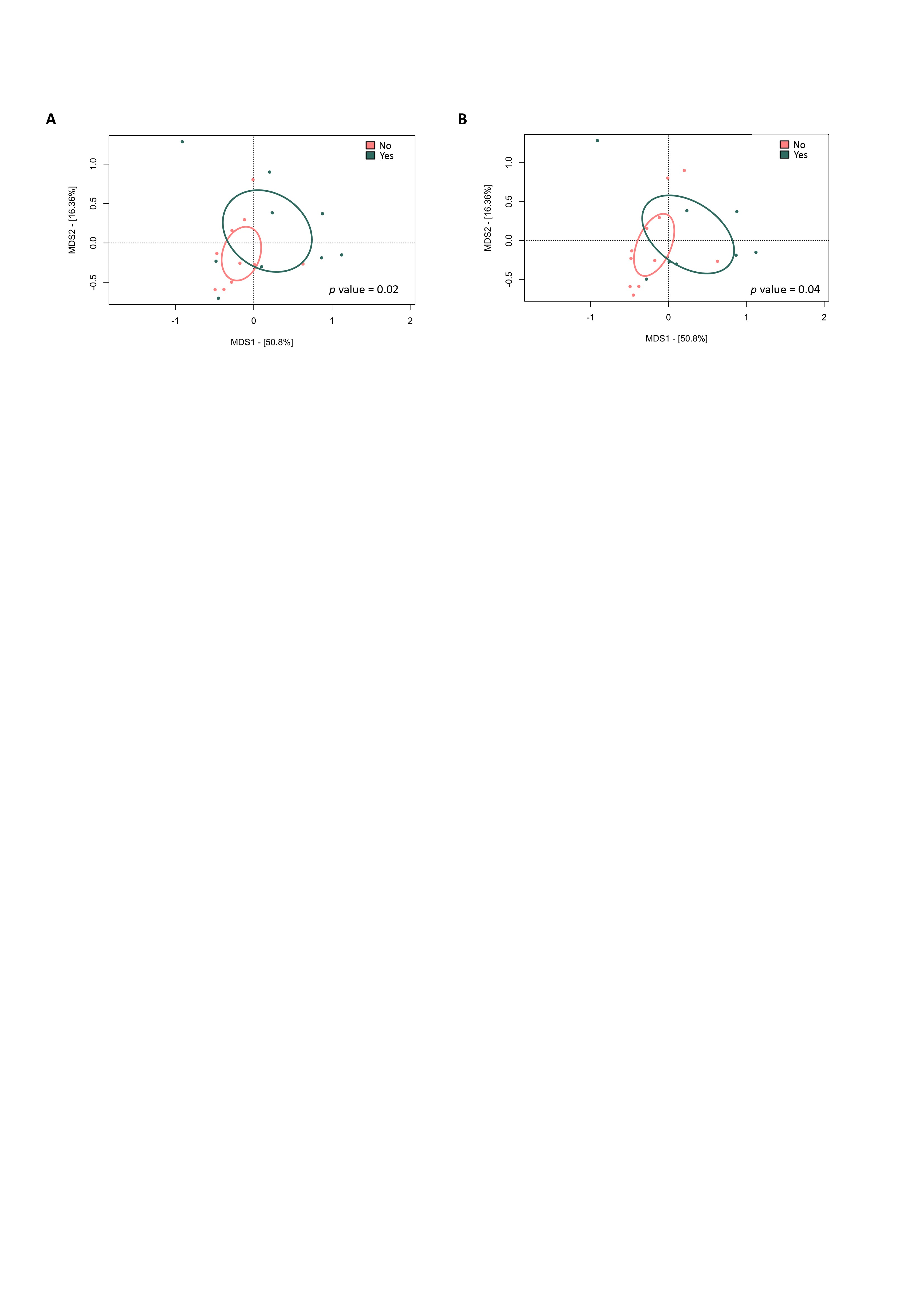


**Figure S1: Impact of confounding factors on the baseline nasopharyngeal microbiota in infants with RSV bronchiolitis.** PCoA based on weighted UniFrac distances between the microbiota profiles of nasopharyngeal samples collected at emergency room admission from patients stratified by antibiotic intake (yes vs. no, left) and cortisone administration (yes vs. no, right). A significant separation between groups was found in both cases (PERMANOVA, *p* ≤ 0.04).


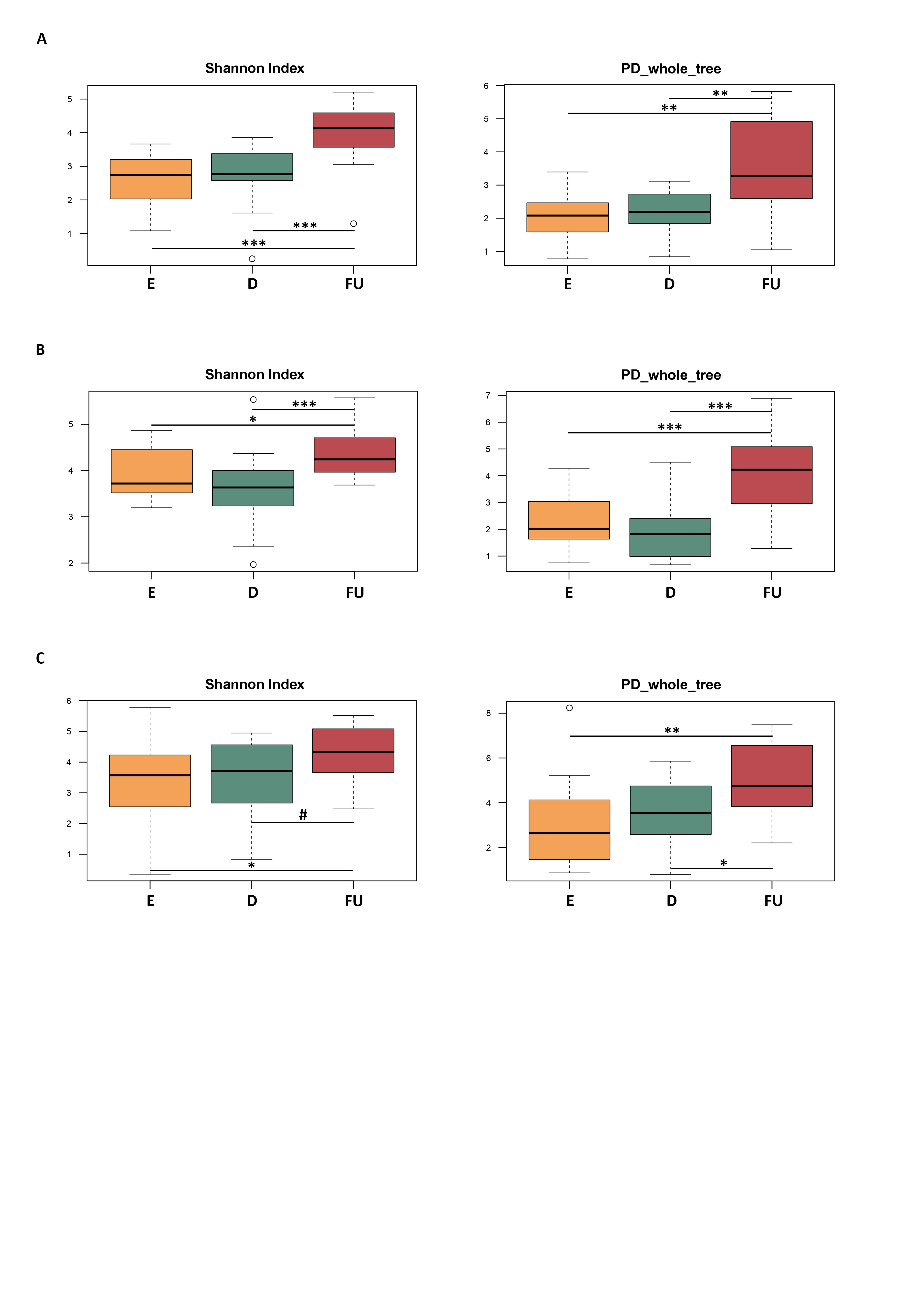


**Figure S2: Alpha diversity dynamics of the gut, oral and nasopharyngeal microbiota in infants with RSV bronchiolitis from emergency room admission to six months after discharge.** Boxplots showing the distribution of alpha diversity, estimated with the Shannon index (left) and Faith’s Phylogenetic Diversity (PD_whole_tree; right), for fecal samples (**A**), oral swabs (**B**) and nasopharyngeal aspirates (**C**) collected at the emergency room admission (E), discharge (D) and six-month follow-up (FU). Wilcoxon test, * *p* value ≤ 0.05; ** *p* value ≤ 0.01; *** *p* value ≤ 0.001; *# p* value ≤ 0.09*.*


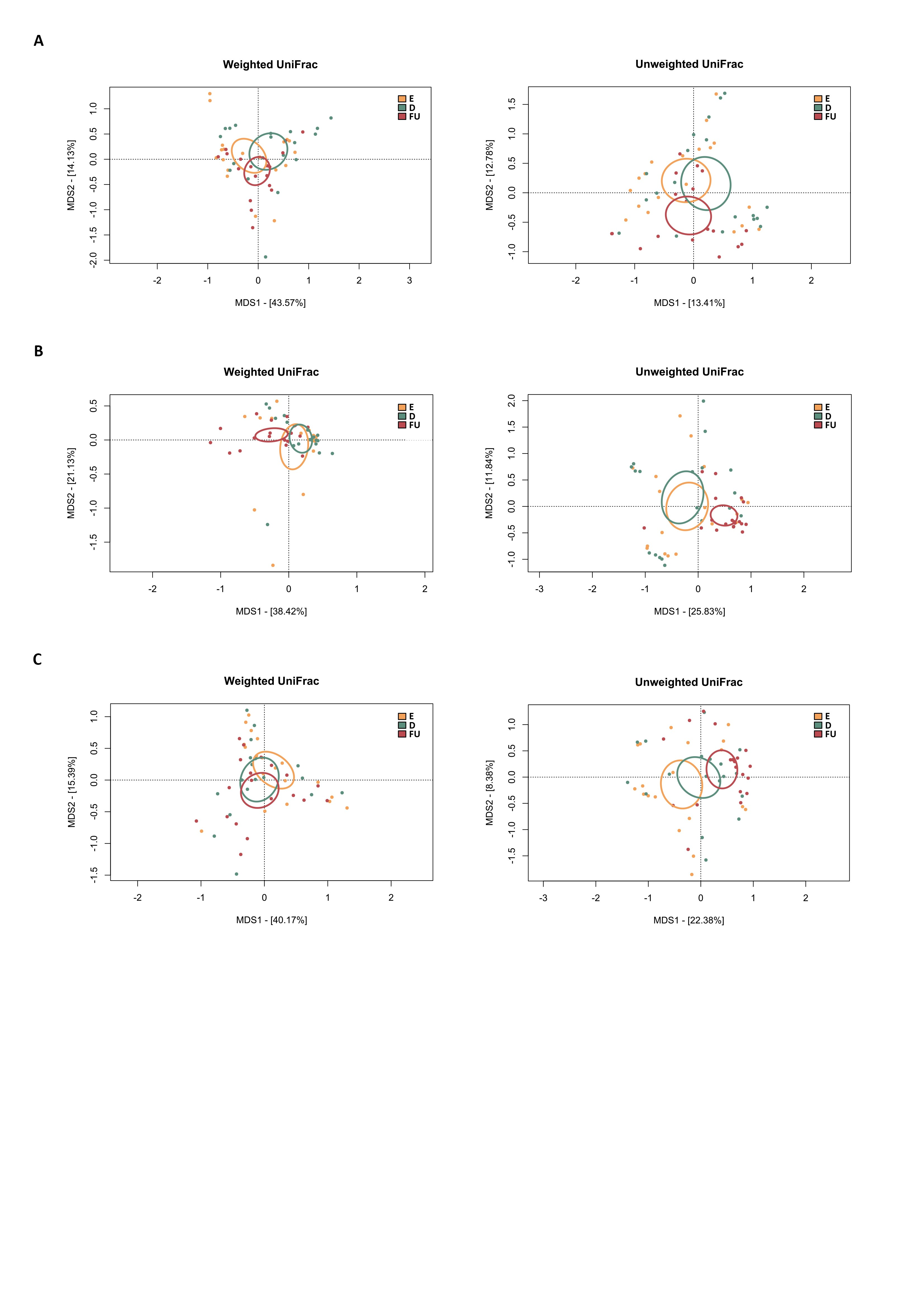


**Figure S3: Beta diversity of the gut, oral and nasopharyngeal microbiota in infants with RSV bronchiolitis from emergency room admission to six months after discharge.** Principal Coordinates Analysis (PCoA) based on weighted UniFrac (left) and unweighted UniFrac (right) distances between gut (**A**), oral (**B**) and nasopharyngeal (**C**) microbiota profiles at the emergency room admission (E), discharge (D) and six-month follow-up (FU). A significant segregation was found between FU samples and those collected at previous timepoints in unweighted UniFrac-based PCoA for all ecosystems (PERMANOVA, *p* ≤ 0.05). In addition, a separation was detected between D and FU oral microbiota profiles in weighted UniFrac-based PCoA (*p* = 0.01).


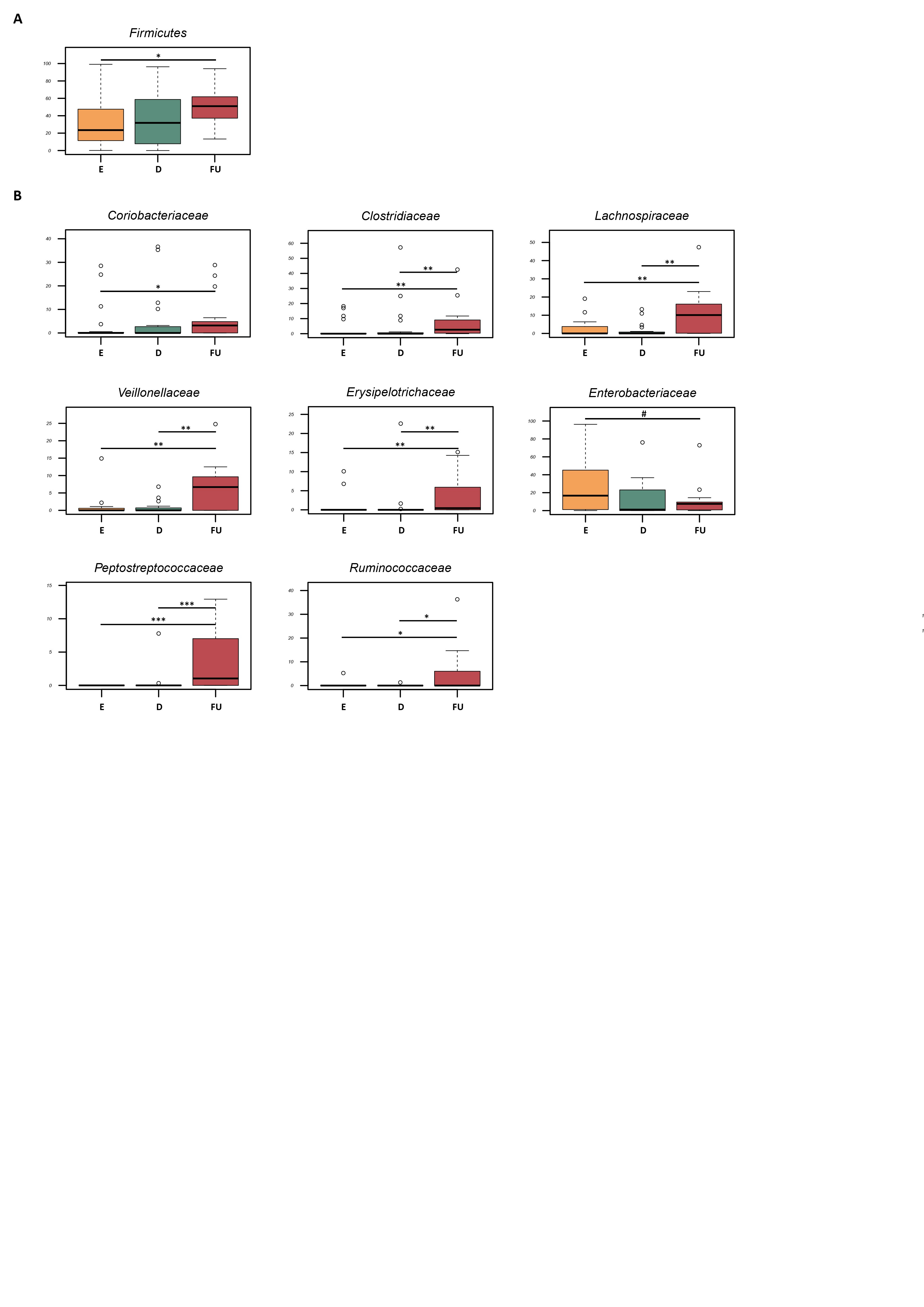


**Figure S4: Gut microbiota phyla and families differentially represented in infants with RSV bronchiolitis from emergency room admission to six months after discharge.** Boxplots showing the relative abundance distribution of phyla (**A**) and families (**B**) of the gut microbiota differentially represented over time. E, emergency room admission; D, discharge; FU, six-month follow-up. Wilcoxon test, * *p* value ≤ 0.05; ** *p* value ≤ 0.01; *** *p* value ≤ 0.001; # *p* value ≤ 0.09.


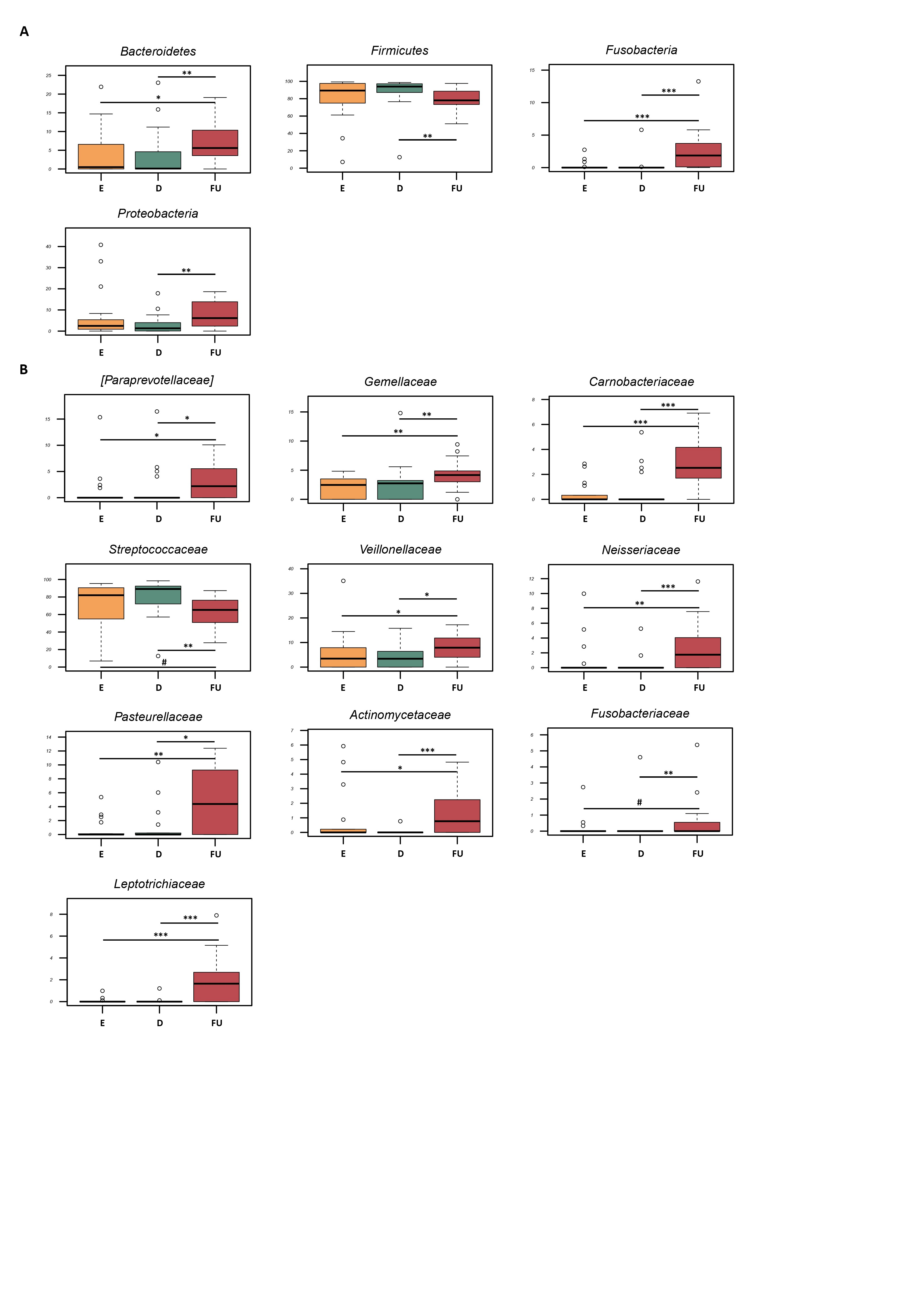


**Figure S5: Oral microbiota phyla and families differentially represented in infants with RSV bronchiolitis from emergency room admission to six months after discharge.** Boxplots showing the relative abundance distribution of phyla (**A**) and families (**B**) of the oral microbiota differentially represented over time. E, emergency room admission; D, discharge; FU, six-month follow-up. Wilcoxon test, * *p* value ≤ 0.05; ** *p* value ≤ 0.01; *** *p* value ≤ 0.001; # *p* value ≤ 0.09.


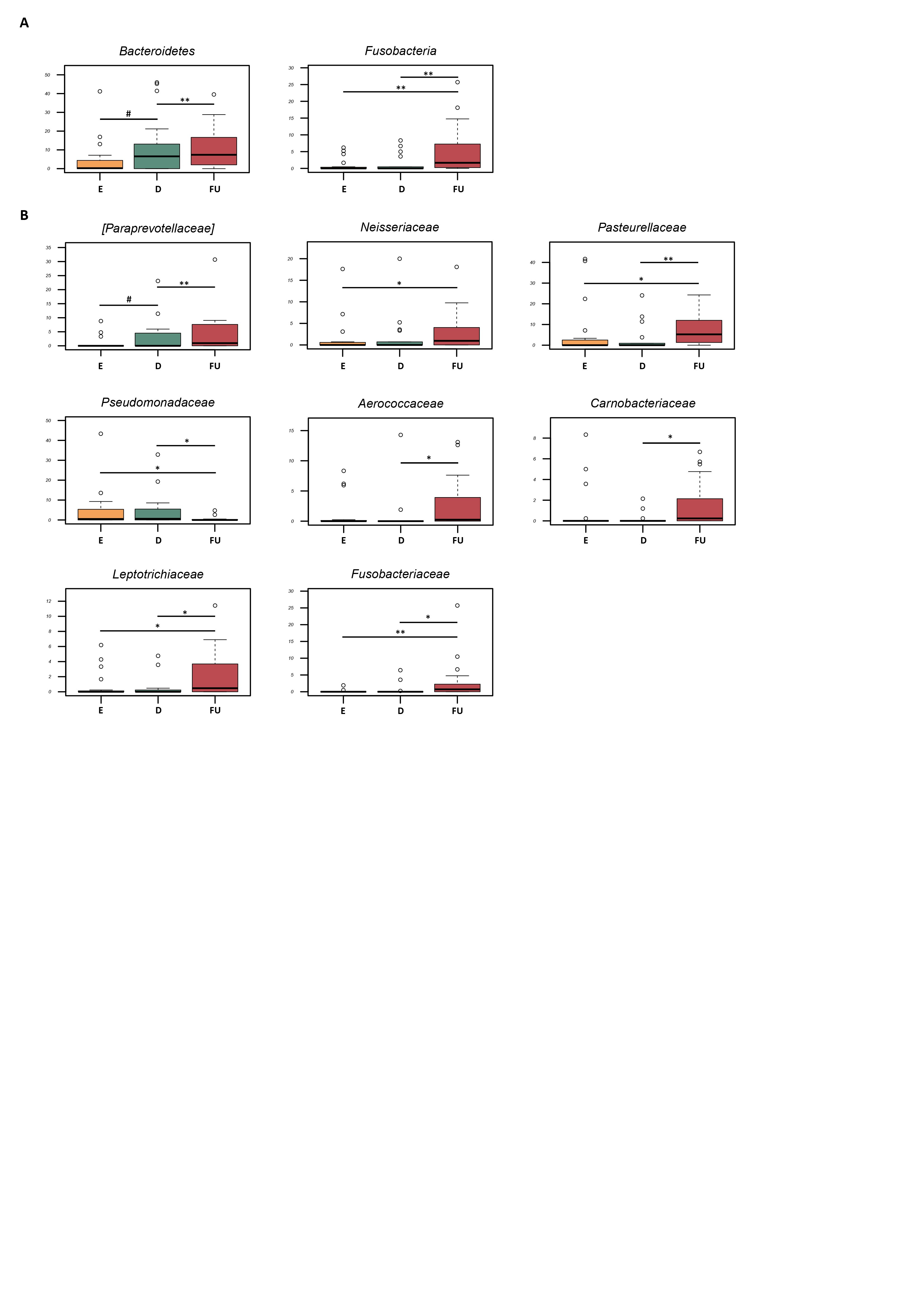


**Figure S6: Nasopharyngeal microbiota phyla and families differentially represented in infants with RSV bronchiolitis from emergency room admission to six months after discharge.** Boxplots showing the relative abundance distribution of phyla (**A**) and families (**B**) of the nasopharyngeal microbiota differentially represented over time. E, emergency room admission; D, discharge; FU, six-month follow-up. Wilcoxon test, * *p* value ≤ 0.05; ** *p* value ≤ 0.01; # *p* value ≤ 0.09.


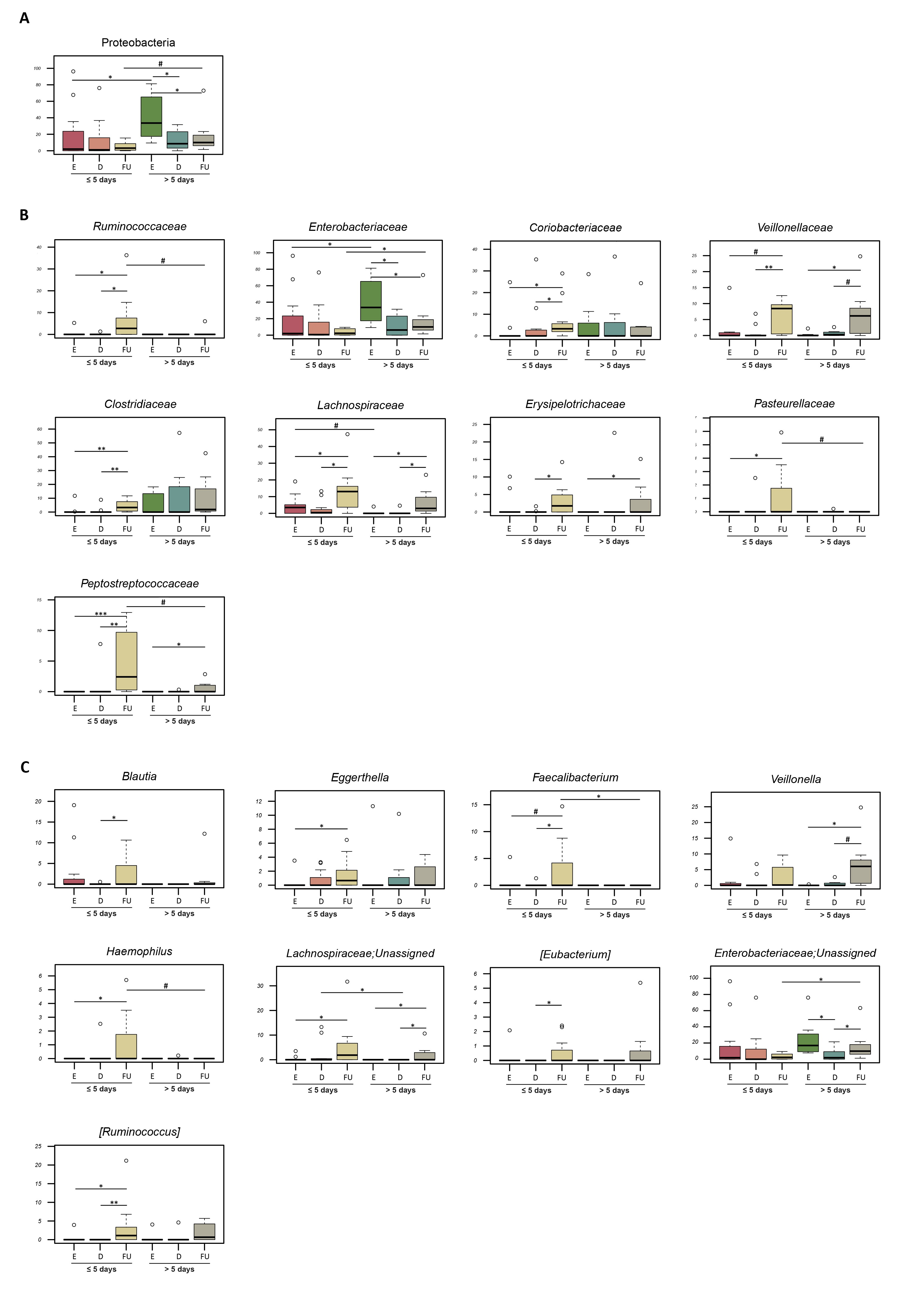


**Figure S7: Differences in gut microbiota composition between infants with RSV bronchiolitis with different length of hospitalization.** Boxplots showing the relative abundance distribution of phyla (**A**), families (**B**) and genera (**C**) differentially represented between patients hospitalized for less than or equal to 5 days vs. more than 5 days, and over time within each patient group. E, emergency room admission; D, discharge; FU, six-month follow-up. Wilcoxon test, * *p* value ≤ 0.05; ** *p* value ≤ 0.01; *** *p* value ≤ 0.001; # *p* value ≤ 0.09.


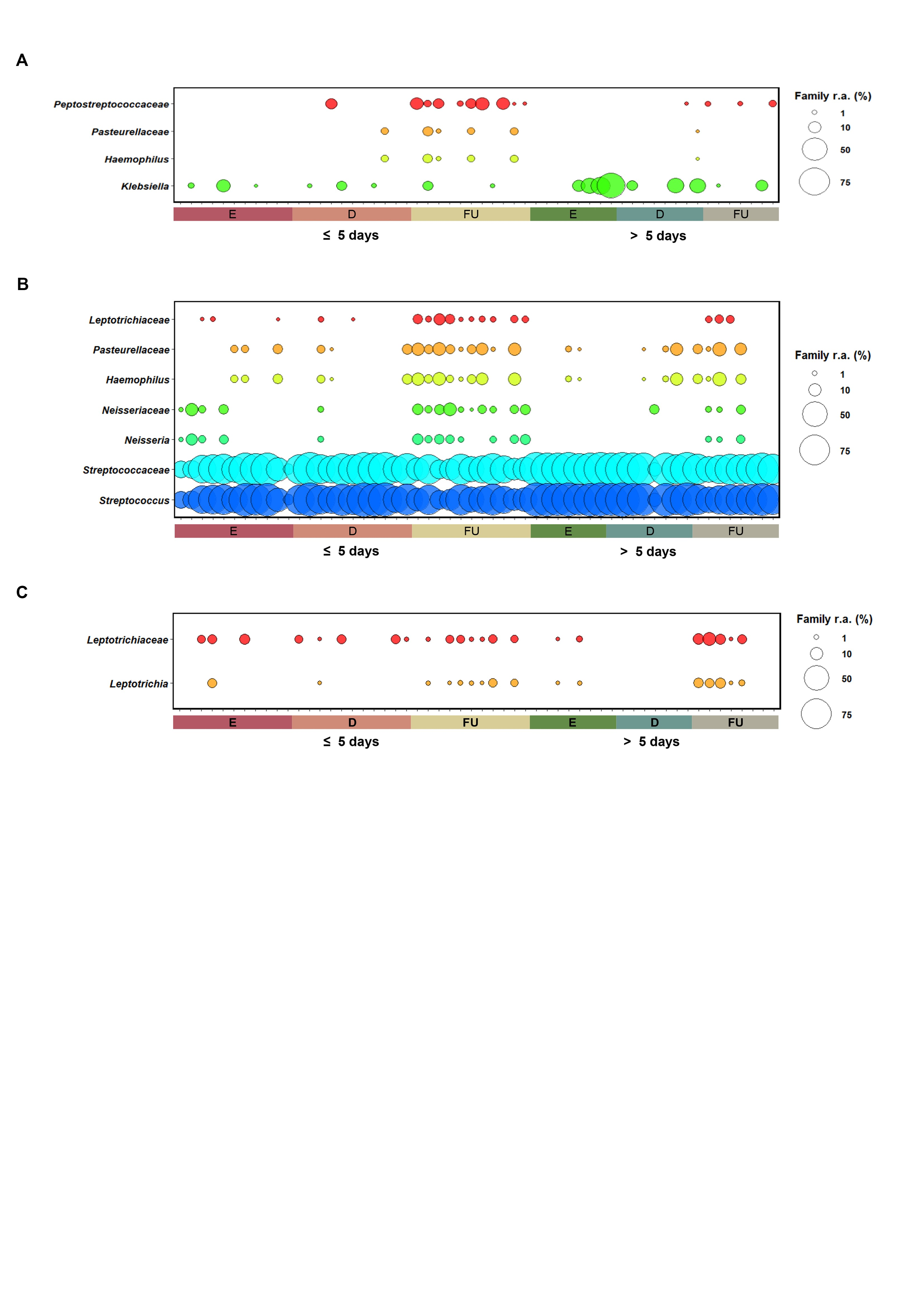
**Figure S8: Relative abundance dynamics of bacterial families and genera with differential distribution in infants with RSV bronchiolitis with different length of hospitalization according to MaAsLin2.** Bubble plot showing the relative abundance distribution of bacterial families and genera differentially represented between patients hospitalized for less than or equal to 5 days and those hospitalized for more than 5 days according to the MaAsLin2 model for each ecosystem (**A**, gut microbiota; **B**, oral microbiota; **C**, nasopharyngeal microbiota). The size of the bubbles represents the percentage of relative abundance according to the legend on the right. E, emergency room admission; D, discharge; FU, six-month follow-up; r. a., relative abundance.


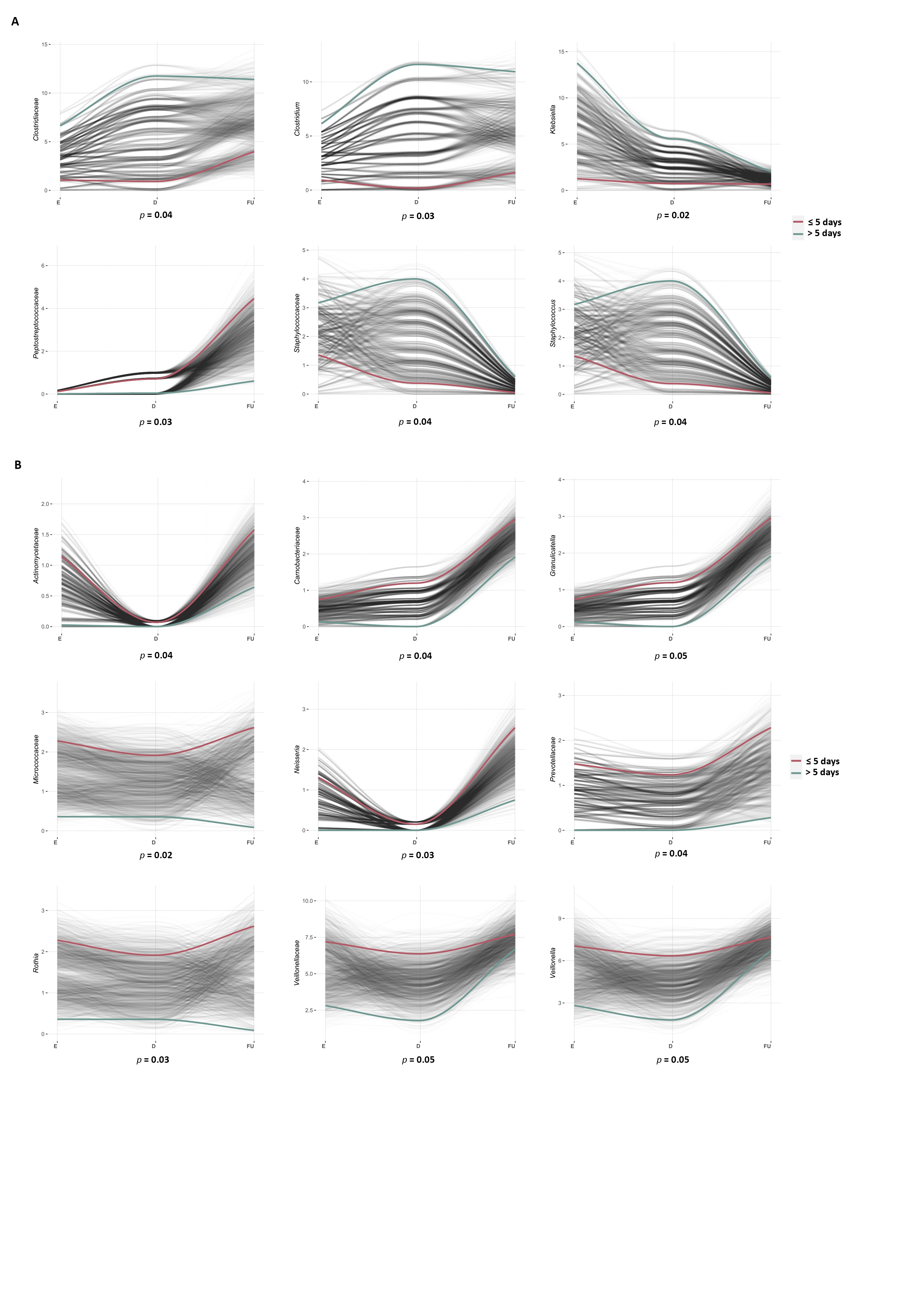


**Figure S9: Statistically significant permuted spline test discriminating infants with RSV bronchiolitis with different length of hospitalization.** Permuspliner output plots showing the relative abundance distribution of taxa in fecal samples (**A**) and oral samples (**B**) distinguishing infants hospitalized for less than or equal to 5 days (group spline in red) from infants hospitalized for more than 5 days (group spline in green) over time. The grey splines show the trends for the permuted data points, with 999 permutations computed, representing the background variance in the data. The *p* value below each plot indicates the result of the permuspliner permutational testing. E, emergency room admission; D, discharge; FU, six-month follow-up.


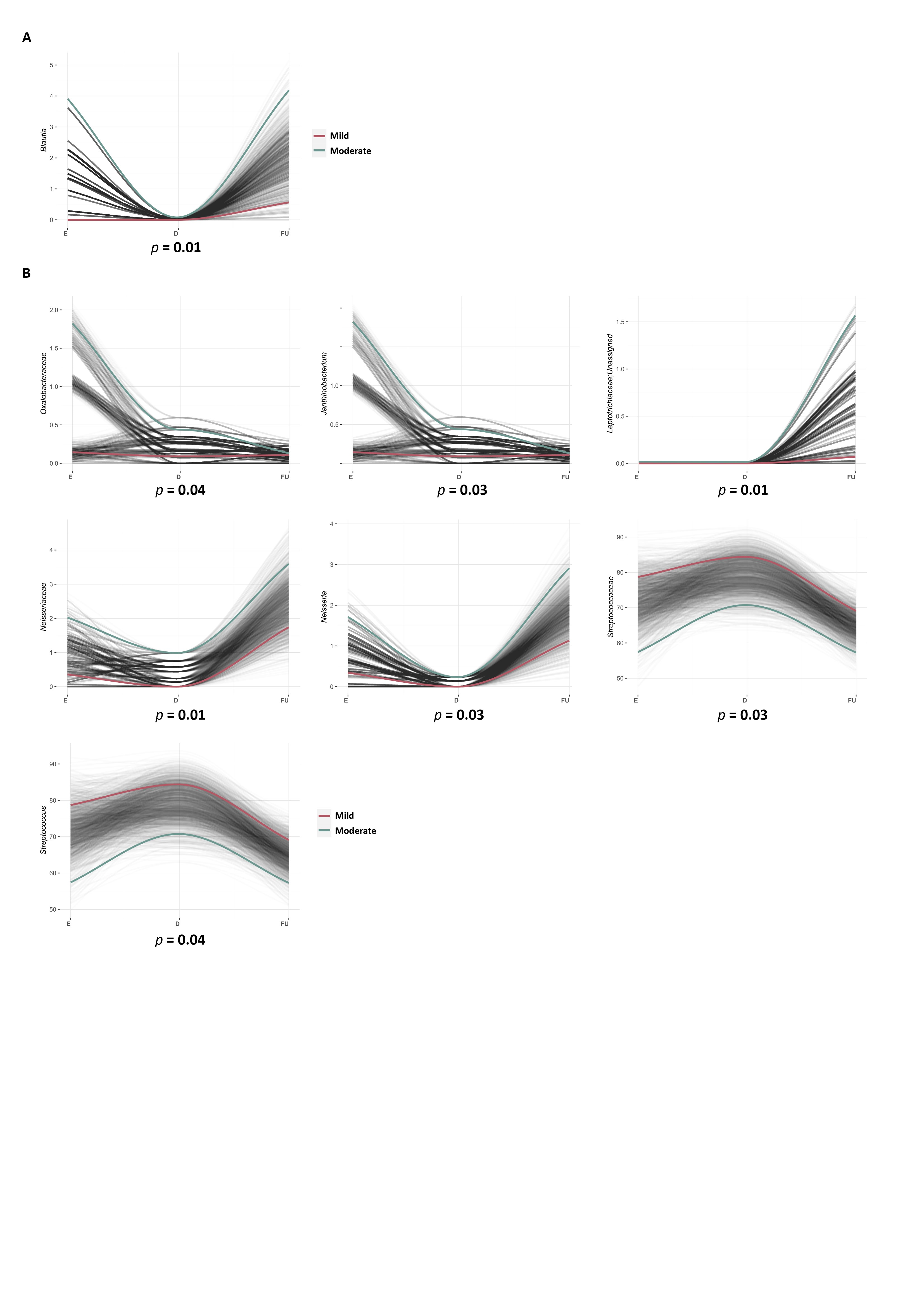


**Figure S10: Statistically significant permuted spline test discriminating infants with RSV bronchiolitis according to clinical infection score.** Permuspliner output plots showing the relative abundance distribution of taxa in fecal samples (**A**) and oral samples (**B**) distinguishing infants according to the clinical infection score (mild: group spline in red; moderate: group spline in green) over time. The grey splines show the trends for the permuted data points, with 999 permutations computed, representing the background variance in the data. The *p* value below each plot indicates the result of the permuspliner permutational testing. E, emergency room admission; D, discharge; FU, six-month follow-up.


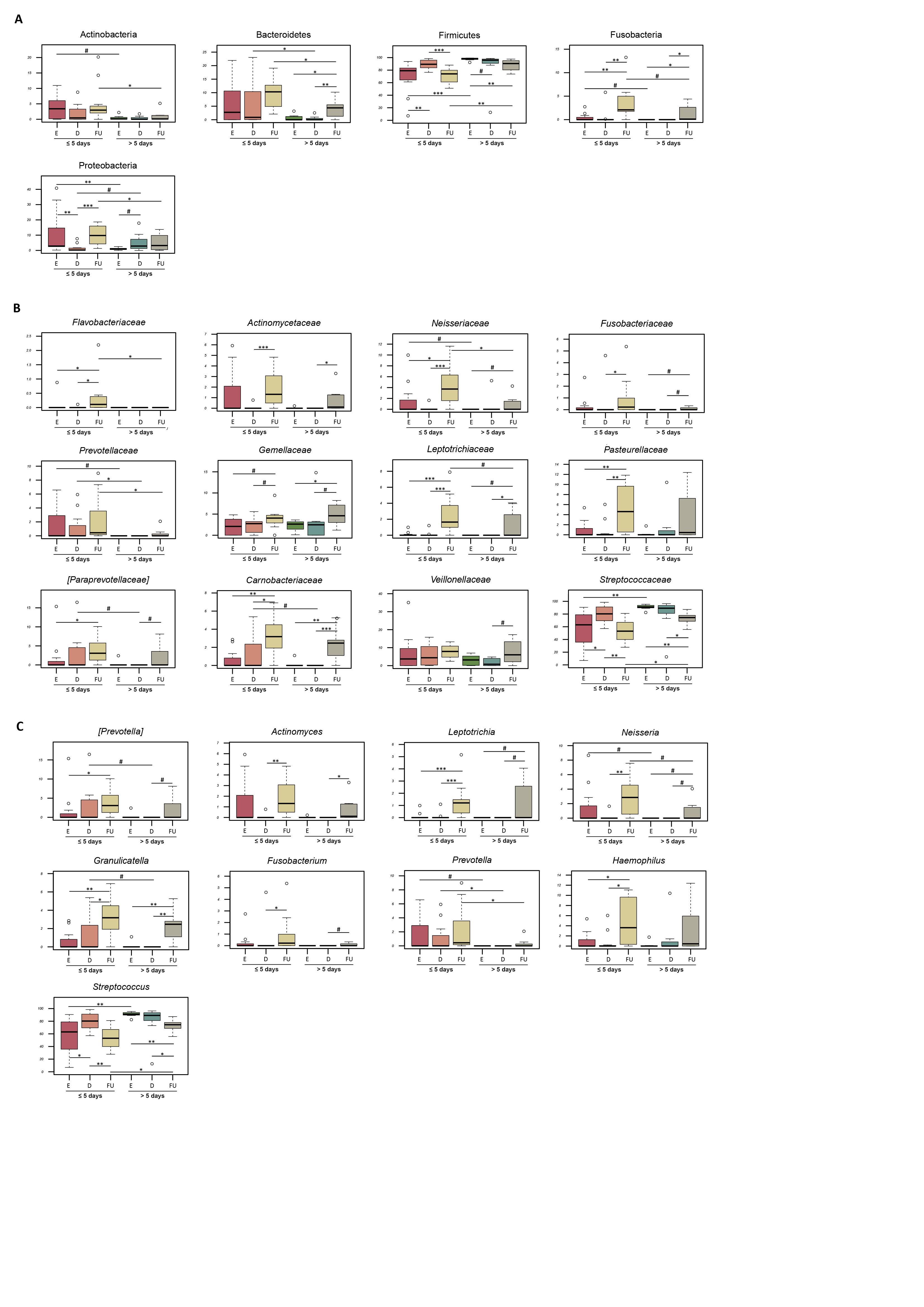


**Figure S11: Differences in oral microbiota composition between infants with RSV bronchiolitis with different length of hospitalization.** Boxplots showing the relative abundance distribution of phyla (**A**), families (**B**) and genera (**C**) differentially represented between patients hospitalized for less than or equal to 5 days vs. more than 5 days, and over time within each patient group. E, emergency room admission; D, discharge; FU, six-month follow-up. Wilcoxon test, * *p* value ≤ 0.05; ** *p* value ≤ 0.01; *** *p* value ≤ 0.001; # *p* value ≤ 0.09.


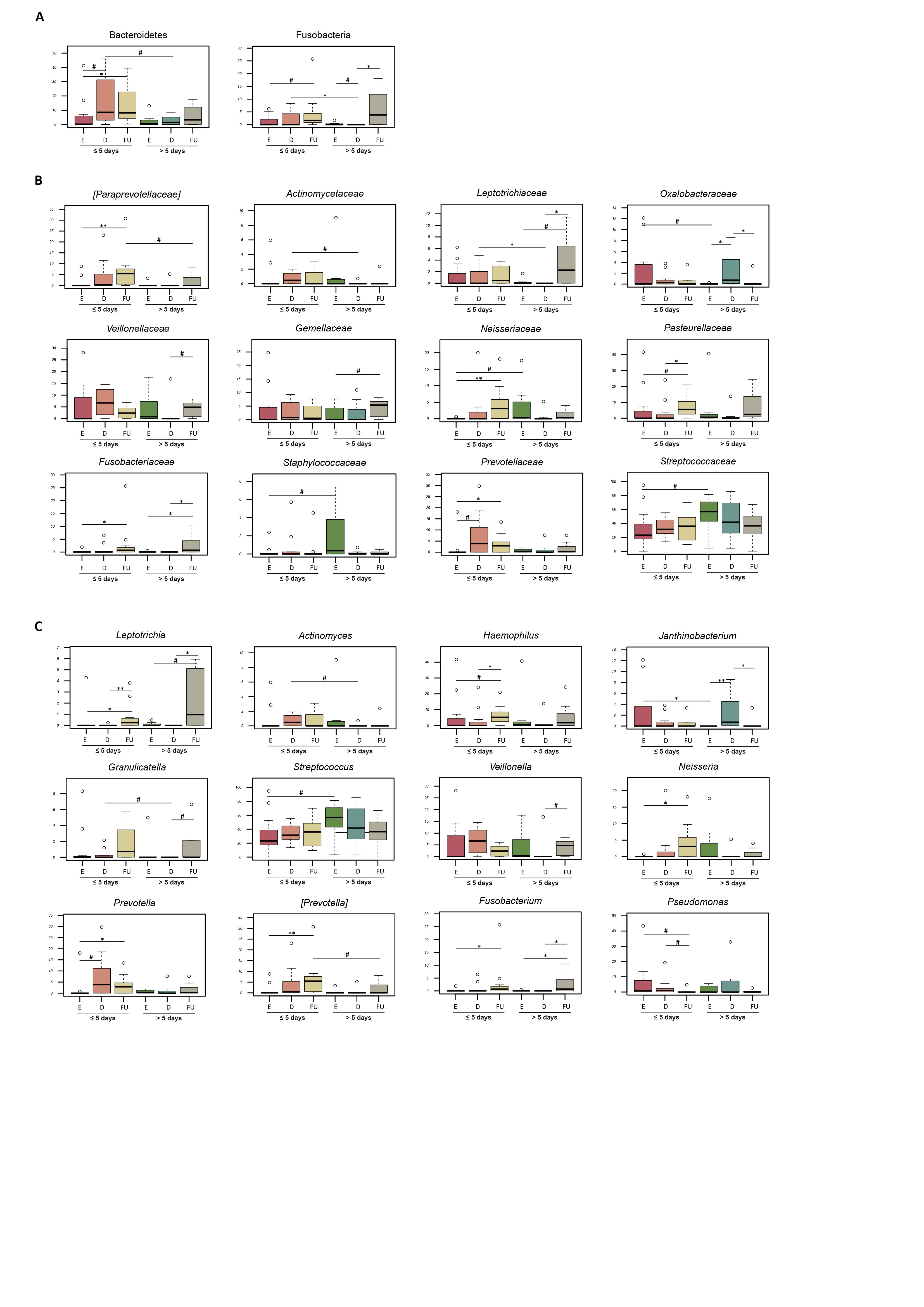


**Figure S12: Differences in nasopharyngeal microbiota composition between infants with RSV bronchiolitis with different length of hospitalization.** Boxplots showing the relative abundance distribution of phyla (**A**), families (**B**) and genera (**C**) differentially represented between patients hospitalized for less than or equal to 5 days vs. more than 5 days, and over time within each patient group. E, emergency room admission; D, discharge; FU, six-month follow-up. Wilcoxon test, * *p* value ≤ 0.05; ** *p* value ≤ 0.01; # *p* value ≤ 0.09.


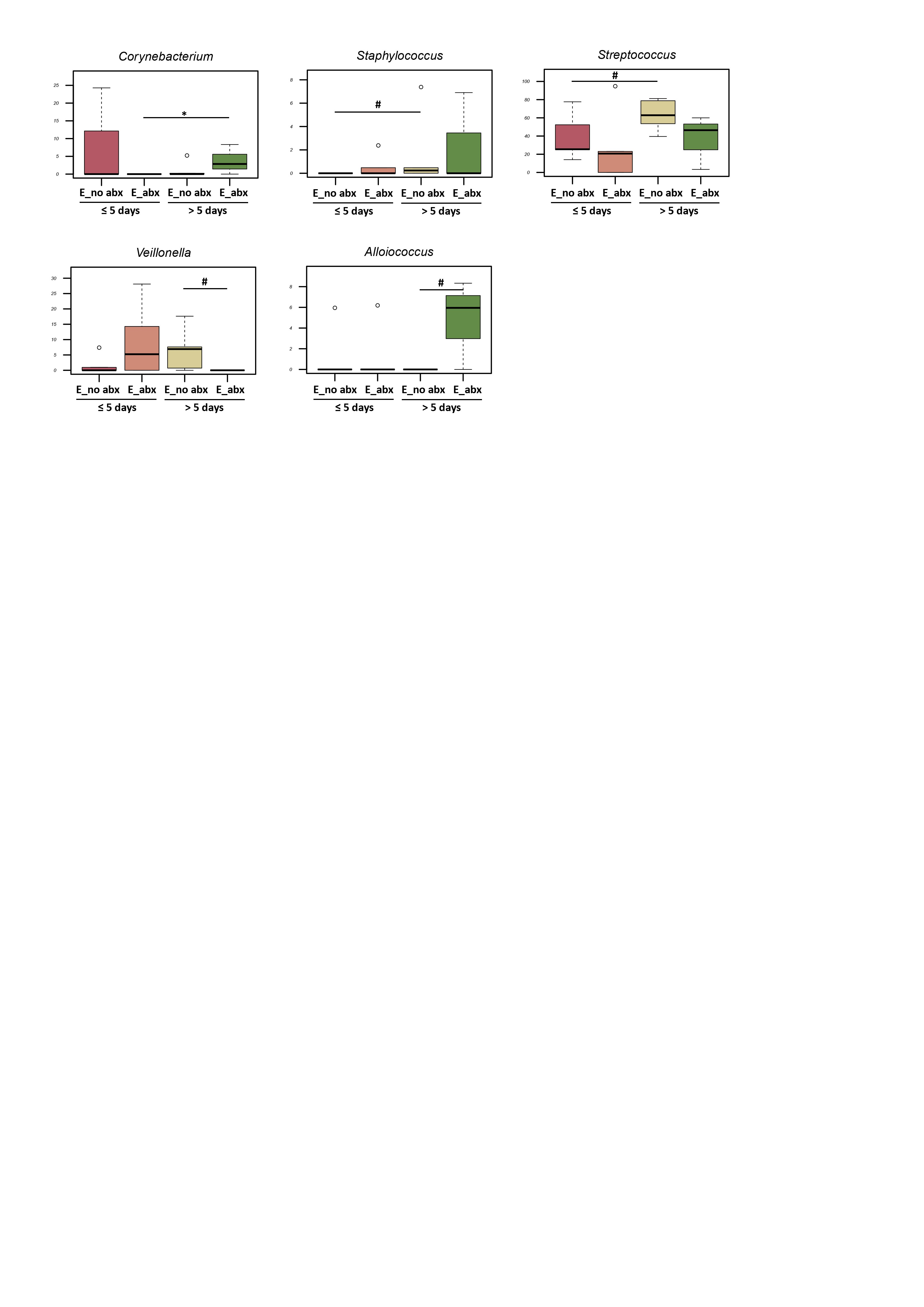


**Figure S13: Differences in nasopharyngeal microbiota composition between infants with RSV bronchiolitis with different length of hospitalization and different antibiotic exposure.** Boxplots showing the relative abundance distribution of differentially represented genera at enrolment (E) between patients hospitalized for less than or equal to 5 days vs. more than 5 days with different antibiotic exposure (no_abx = no antibiotic administration at emergency room admission, abx = antibiotic administration at emergency room admission). Wilcoxon test, * *p* value ≤ 0.05; # *p* value ≤ 0.09.

**Table S1: Statistically significant associations obtained by MaAsLin2.** Table summarizing the significant associations between microbial taxa, timepoints and condition (*i.e.*, infants hospitalized for less than or equal to 5 days vs. more than 5 days) per ecosystem, obtained by applying MaAsLin2 R. E, emergency room admission; D, discharge; FU, six-month follow-up.

| **Ecosystem** | **Taxa** | **Timepoint/Condition** | **Coefficient** | ***p* value** |
| --- | --- | --- | --- | --- |
| Gut | *Veillonellaceae* | FU | 5.39 | 0.0003 |
| Gut | *Peptostreptococcaceae* | FU | 3.38 | 0.0006 |
| Gut | *Lachnospiraceae* | FU | 8.14 | 0.002 |
| Gut | *Enterobacteriaceae* | FU | -19.03 | 0.006 |
| Gut | *Enterobacteriaceae* | D | -16.25 | 0.02 |
| Gut | *Peptostreptococcaceae* | FU in infants hospitalized for ≤ 5 days | 6.64 | 0.00003 |
| Gut | *Pasteurellaceae* | FU in infants hospitalized for ≤ 5 days | 1.75 | 0.0014 |
| Gut | *Haemophilus* | FU in infants hospitalized for ≤ 5 days | 1.72 | 0.0014 |
| Gut | *Klebsiella* | E in infants hospitalized for > 5 days | 15.9 | 0.0008 |
| Oral | *Carnobacteriaceae* | FU | 2.41 | 1.54E-06 |
| Oral | *Leptotrichiaceae* | FU | 1.92 | 2.82E-05 |
| Oral | *Pasteurellaceae* | FU | 3.74 | 0.001 |
| Oral | *Gemellaceae* | FU | 2.29 | 0.008 |
| Oral | *Leptotrichiaceae* | FU in infants hospitalized for ≤ 5 days | 2.84 | 0.0002 |
| Oral | *Haemophilus* | FU in infants hospitalized for ≤ 5 days | 6.84 | 0.0001 |
| Oral | *Pasteurellaceae* | FU in infants hospitalized for ≤ 5 days | 7.49 | 4.19E-05 |
| Oral | *Streptococcaceae* | E in infants hospitalized for > 5 days | 31.3 | 0.003 |
| Oral | *Streptococcus* | E in infants hospitalized for > 5 days | 31.3 | 0.003 |
| Oral | *Neisseriaceae* | FU in infants hospitalized for ≤ 5 days | 3.83 | 0.005 |
| Oral | *Neisseria* | FU in infants hospitalized for ≤ 5 days | 2.87 | 0.006 |
| Nasopharyngeal | *Leptotrichiaceae* | FU in infants hospitalized for > 5 days | 7.18 | 2.29E-06 |
| Nasopharyngeal | *Leptotrichiaceae* | FU in infants hospitalized for ≤ 5 days | 6.51 | 7.77E-05 |
| Nasopharyngeal | *Leptotrichia* | FU in infants hospitalized for > 5 days | 3.82 | 3.74E-05 |
| Nasopharyngeal | *Leptotrichia* | FU in infants hospitalized for ≤ 5 days | 3.14 | 0.002 |

**Table S2: Statistically significant *p* values from PERMANOVA tests.** Table summarizing the significant *p* values (*p* ≤ 0.05) obtained from PERMANOVA tests performed on Principal Coordinates Analysis (PCoA) based on weighted UniFrac and unweighted UniFrac distances between microbiota profiles in the gut, oral and nasopharyngeal ecosystem (related to Figures 3B, 4B, and 5B). E, emergency room admission; D, discharge; FU, six-month follow-up.

| **Ecosystem** | **Metric** | **Comparison** | ***p* value** |
| --- | --- | --- | --- |
| Gut | Unweighted UniFrac | D vs FU in infants hospitalized for ≤ 5 days | 0.026 |
| Oral | Weighted UniFrac | E in infants hospitalized for ≤ 5 days vs. E in infants hospitalized for > 5 days | 0.003 |
| Oral | Weighted UniFrac | D vs. FU in infants hospitalized for ≤ 5 days | 0.017 |
| Oral | Weighted UniFrac | FU in infants hospitalized for ≤ 5 days vs. FU in infants hospitalized for > 5 days | 0.017 |
| Oral | Weighted UniFrac | E vs. FU in infants hospitalized for > 5 days | 0.001 |
| Oral | Weighted UniFrac | D vs. FU in infants hospitalized for > 5 days | 0.04 |
| Oral | Unweighted UniFrac | E vs. FU in infants hospitalized for ≤ 5 days | 0.005 |
| Oral | Unweighted UniFrac | D vs. FU in infants hospitalized for ≤ 5 days | 0.001 |
| Oral | Unweighted UniFrac | D in infants hospitalized for ≤ 5 days vs. D in infants hospitalized for > 5 days | 0.046 |
| Oral | Unweighted UniFrac | FU in infants hospitalized for ≤ 5 days vs. FU in in infants hospitalized for > 5 days | 0.015 |
| Oral | Unweighted UniFrac | D vs. FU in infants hospitalized for > 5 days | 0.006 |
| Nasopharyngeal | Unweighted UniFrac | E vs. D in infants hospitalized for ≤ 5 days | 0.015 |
| Nasopharyngeal | Unweighted UniFrac | E vs. FU in infants hospitalized for ≤ 5 days | 0.0005 |
| Nasopharyngeal | Unweighted UniFrac | D vs. FU in infants hospitalized for > 5 days | 0.046 |
